# A quick and innovative pipeline for producing chondrocyte-homing peptide-modified extracellular vesicles by three-dimensional dynamic culture of hADSCs spheroids to modulate the fate of remaining ear chondrocytes in the M1 macrophage-infiltrated microenvironment

**DOI:** 10.1186/s12951-024-02567-5

**Published:** 2024-05-30

**Authors:** Jianguo Chen, Enchong Zhang, Yingying Wan, Tianyu Huang, Yuchen Wang, Haiyue Jiang

**Affiliations:** 1 Chinese Academy of Medical Sciences & Peking Union Medical College Plastic Surgery Hospital and Institute, Shijingshan District, Beijing, 100144 China; 2 Department of Urology, Shengjing Hospital of China Medical University, No. 36, Sanhao Street, Heping District, Shenyang, 110004 China; 3 DongFang Hospital of Beijing University of Chinese Medicine, Fengtai District, Beijing, 100078 China

**Keywords:** Adipose-derived mesenchymal stem cells, Chondrocyte homing peptide, Extracellular vesicles, M1 macrophage, Remaining ear chondrocytes

## Abstract

**Background:**

Extracellular vesicles (EVs) derived from human adipose-derived mesenchymal stem cells (hADSCs) have shown great therapeutic potential in plastic and reconstructive surgery. However, the limited production and functional molecule loading of EVs hinder their clinical translation. Traditional two-dimensional culture of hADSCs results in stemness loss and cellular senescence, which is unfavorable for the production and functional molecule loading of EVs. Recent advances in regenerative medicine advocate for the use of three-dimensional culture of hADSCs to produce EVs, as it more accurately simulates their physiological state. Moreover, the successful application of EVs in tissue engineering relies on the targeted delivery of EVs to cells within biomaterial scaffolds.

**Methods and Results:**

The hADSCs spheroids and hADSCs gelatin methacrylate (GelMA) microspheres are utilized to produce three-dimensional cultured EVs, corresponding to hADSCs spheroids-EVs and hADSCs microspheres-EVs respectively. hADSCs spheroids-EVs demonstrate excellent production and functional molecule loading compared with hADSCs microspheres-EVs. The upregulation of eight miRNAs (i.e. hsa-miR-486-5p, hsa-miR-423-5p, hsa-miR-92a-3p, hsa-miR-122-5p, hsa-miR-223-3p, hsa-miR-320a, hsa-miR-126-3p, and hsa-miR-25-3p) and the downregulation of hsa-miR-146b-5p within hADSCs spheroids-EVs show the potential of improving the fate of remaining ear chondrocytes and promoting cartilage formation probably through integrated regulatory mechanisms. Additionally, a quick and innovative pipeline is developed for isolating chondrocyte homing peptide-modified EVs (CHP-EVs) from three-dimensional dynamic cultures of hADSCs spheroids. CHP-EVs are produced by genetically fusing a CHP at the N-terminus of the exosomal surface protein LAMP2B. The CHP + LAMP2B-transfected hADSCs spheroids were cultured with wave motion to promote the secretion of CHP-EVs. A harvesting method is used to enable the time-dependent collection of CHP-EVs. The pipeline is easy to set up and quick to use for the isolation of CHP-EVs. Compared with nontagged EVs, CHP-EVs penetrate the biomaterial scaffolds and specifically deliver the therapeutic miRNAs to the remaining ear chondrocytes. Functionally, CHP-EVs show a major effect on promoting cell proliferation, reducing cell apoptosis and enhancing cartilage formation in remaining ear chondrocytes in the M1 macrophage-infiltrated microenvironment.

**Conclusions:**

In summary, an innovative pipeline is developed to obtain CHP-EVs from three-dimensional dynamic culture of hADSCs spheroids. This pipeline can be customized to increase EVs production and functional molecule loading, which meets the requirements for regulating remaining ear chondrocyte fate in the M1 macrophage-infiltrated microenvironment.

**Graphical Abstract:**

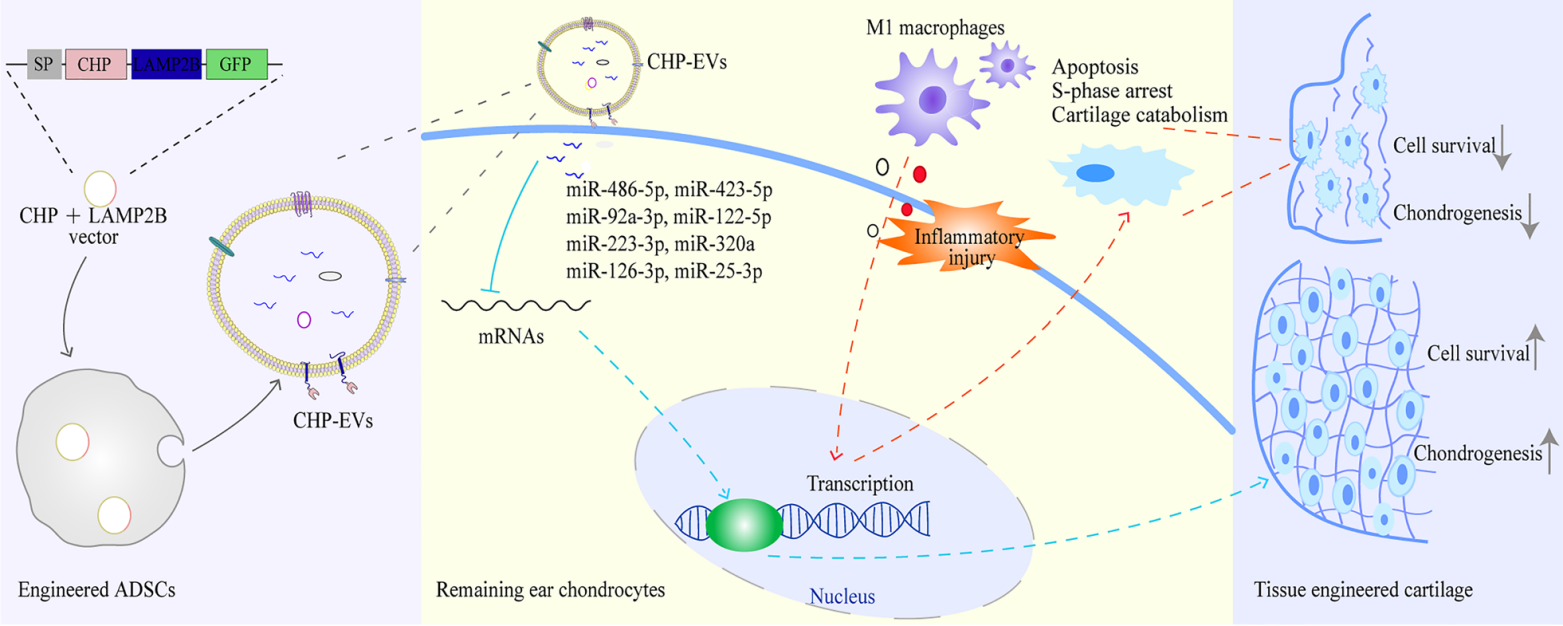

**Supplementary Information:**

The online version contains supplementary material available at 10.1186/s12951-024-02567-5.

## Introduction

Microtia has a significant impact on both the aesthetic appearance and psychological well-being of affected children. The current standard method for ear reconstruction in microtia involves autologous rib cartilage ear framework transplantation. However, this procedure is time-consuming, taking around 3 months to complete, and is costly, posing challenges for the child’s education and placing financial strain on families and society. Moreover, harvesting rib cartilage can lead to donor site damage and chest wall deformities. The intricate process of sculpting the ear framework requires a high level of expertise, making it difficult to train new doctors in this technique. Fortunately, recent advancements in materials science, three-dimensional printing, and tissue engineering technology have opened up new possibilities for creating tissue-engineered ear frameworks for clinical applications. Tissue-engineered ears offer great hope for children with microtia. Since 2018, our ear reconstruction center achieved a significant milestone by successfully conducting a clinical trial using tissue-engineered ears for external ear reconstruction, making this the first international achievement in this field [1]. However, the tissue-engineered ears implanted in the body exhibited varying levels of deformation and collapse in later stages due to inadequate synthesis of mature cartilage matrix by remaining ear chondrocytes. Therefore, it is crucial to investigate the reasons for the inadequate synthesis of tissue-engineered cartilage matrix and explore potential intervention strategies.

Macrophage can be classified into two types: tissue-resident macrophage and macrophage derived from circulating monocytes, which plays crucial roles in maintaining tissue balance and promoting tissue regeneration after injury. Macrophage can differentiate into at least two subgroups with distinct immune functions based on various stimuli. The M1 subgroup, known as classically activated macrophage, mainly contributes to inflammatory responses, while the M2 subgroup, known as alternatively activated macrophage, is primarily involved in wound healing, inflammation suppression, and tissue repair [2]. M1 macrophage plays a significant role in the sterile inflammatory response caused by tissue-engineered cartilage. Studies have revealed that various factors, including synthetic biocompatible materials and their degradation products, the condition of chondrocytes (such as chondrocyte aging and products, CD47 conformational changes after chondrocyte passage culture/ectopic culture), and the stimulation of chondrocyte extracellular matrix degradation products, can cause the infiltration of M1 macrophage [3–5]. The infiltration of M1 macrophage will release proinflammatory mediators such as IL-6, IL-1β and extracellular vesicles, inhibiting the synthesis of cartilage matrix [4, 6, 7]. Therefore, it is essential to enhance the resistance of remaining auricular chondrocytes against M1 macrophage infiltration to promote the advancement of tissue-engineered cartilage.

Extracellular vesicles (EVs), originating from endosomes, typically have an average diameter of 100 nm. They are released into the extracellular environment and contain various substances such as proteins, lipids, mRNA, miRNA, DNA, and metabolites [8]. EVs serve as communication carriers between cells to alter recipient cell gene expression and function, inheriting the functionality and components of the original cell [9]. In the field of regenerative medicine, the use of mesenchymal stem cell (MSCs)-derived EVs has shown promise in cartilage regeneration. These EVs inherit the stemness characteristics and promote cartilage regeneration [10, 11]. Compared to synthetic drug carriers, EVs have advantages such as low cytotoxicity and immunogenicity, high tissue and cell permeability, and the ability to modulate certain cellular pathways. Specifically, EVs derived from adipose-derived MSCs (ADSCs-EVs) have been found to significantly promote the migration, proliferation, and chondrogenic differentiation of bone marrow MSCs and cartilage regeneration [12]. Compared with other sources of MSCs, human ADSCs (hADSCs) are also easier to obtain clinically, making hADSCs-EVs a more promising option for clinical research on cartilage regeneration. However, thorough investigations on the function of hADSC-EVs in protecting cartilage formation in a microenvironment infiltrated by M1 macrophage are lacking.

The low yield and poor functional molecule loading contribute to limited effectiveness of natural hADSCs-EVs in tissue-engineered cartilage. The production and cargos loading of EVs is affected by different culture conditions [13]. Notably, EVs released from three-dimensional culture closely resemble those obtained from patients, unlike EVs from two-dimensional culture. However, producing EVs by three-dimensional cell culture is challenging. Biological scaffolds such as Matrigel and hydrogel scaffolds have been used for culture, but their gel-like structure makes it difficult to dissolve them and collect encapsulated EVs and cells for analysis [14, 15]. Therefore, there is an urgent need for new three-dimensional culture platform to facilitate the production and isolation of EVs. In this study, we introduce two three-dimensional culture models, hADSCs spheroids and hADSCs gelatin methacrylate (GelMA) microspheres (hADSCs microspheres), in a miniaturized dynamic wave-motion bioreactor to promote production of hADSCs-EVs. hADSCs can easily form cell spheroids within 24 h in ultralow-adhesion well plates with a U-shaped bottom. GelMA microspheres, prepared using optimized emulsion crosslinking technology, retain the excellent bioactivity and degradation characteristics of GelMA. GelMA microspheres have the potential to serve as a unit for single cell culture. This technology can be applied to various research fields such as three-dimensional cell culture, drug delivery, tissue engineering, and regenerative medicine. To collect hADSCs-EVs, we have developed a harvesting method that allows us to monitor the growth and properties of hADSCs spheroids or hADSCs microspheres while simultaneously producing EVs. The conditioned medium is collected every 24 h and then subjected to low-speed centrifugation at 1000 × g for 10 min. This gentle centrifugation has no effect on the hADSCs spheroids or hADSCs microspheres and allows us to isolate EVs from the conditioned medium. The remaining pellet is then reseeded into wells with fresh medium for continued culture. We have thoroughly investigated the characteristics of hADSCs-EVs derived from hADSCs spheroids and hADSCs microspheres, including yield, size distribution, morphology, EVs markers, and miRNA cargos. Furthermore, we have summarized the potential beneficial miRNAs that can regulate the fate of remaining ear chondrocytes and tissue-engineered cartilage in a microenvironment infiltrated by M1 macrophage.

Delivering drugs specifically to chondrocytes is a difficult task due to the dense matrix and negatively charged proteoglycans. Natural hADSCs-EVs lack the ability to target chondrocytes. However, we can enhance its targeted delivery function by expressing targeting peptides on the lipid bilayer membrane. Chondrocyte-homing peptide (CHP), with the sequence of DWRVIIPPRPSA, is a specific targeting peptide for chondrocytes [16]. CHP has been shown to effectively penetrate dense matrices and hydrogels and interact with chondrocytes, regardless of species differences. By conjugating CHP to therapeutic vectors or drugs, specific targeting of chondrocytes can be achieved [17, 18]. In our study, we successfully generate hADSCs-derived CHP-modified EVs (CHP-EVs) by transfecting a plasmid vector carrying the fusion gene of CHP + LAMP2B into hADSCs. Our research presents a fast and innovative pipeline for collecting and isolating CHP-EVs via three-dimensional dynamic culture of hADSCs spheroids. Compared with EVs without CHP surface modification, CHP-EVs have a greater ability to deliver functional miRNAs to remaining ear chondrocytes with targeting capabilities. Overall, CHP-EVs exhibit more significant therapeutic effects in regulating the fare of remaining ear and promoting formation of tissue-engineered cartilage in a microenvironment infiltrated by M1 macrophage. This research is of great social significance and has the potential to significantly improve the outcomes of tissue-engineered cartilage transplantation.

## Materials and methods

### Patient enrollment and ethical approval

The study adhered strictly to the guidelines and ethical principles approved by the Ethics Committee of Plastic Surgery Hospital [2023 (155)]. Prior to enrollment and collection of the pictures and specimens of remaining ear, written informed consent was obtained from their parents of microtia children. Microtia children who underwent ear reconstructive surgery were the source of primary remaining ear chondrocytes and hADSCs.

### Primary remaining ear chondrocytes isolation and culture

The remaining ear cartilage was minced and then incubated in Dulbecco's modified Eagle's medium (DMEM) (Gibco) supplemented with 0.2% type IV collagenase (Sigma-Aldrich) and 1% penicillin-streptomycin (P/S) at 37 °C overnight. The chondrocytes obtained from this process were cultured in a standard humidified incubator (37 °C, 5% CO^2^) in DMEM supplemented with 10% fetal bovine serum (FBS) and 1% P/S [19].

### M1 macrophage culture

The THP-1 human monocyte cell line (Procell, China) was cultured in T25 flasks with RPMI 1640 medium (Solarbio) supplemented with 10% FBS and 1% P/S. To induce the M0 phenotype, the cells were treated with 100 ng/mL phorbol 12-myristate-13-acetate (Sigma-Aldrich). Subsequently, M1 polarization was stimulated by adding 1 mg/mL lipopolysaccharide (Sigma-Aldrich) and 50 ng/mL interferon gamma (SinoBiological) to fresh medium [20].

### Primary hADSCs isolation, culture and characterization

In brief, adipose tissues were finely minced and digested in PBS (Gibco) containing 0.1% type I collagenase (Sigma-Aldrich) at 37 °C. After centrifugation, the lipid layers on top were removed, and the primary cells were suspended and cultured in mesenchymal stem cell medium (ScienCell) in a standard humidified incubator (37 °C, 5% CO^2^). Flow cytometry analysis was performed to determine the phenotype of the hADSCs.

### Culture of hADSCs spheroids and hADSCs microspheres

In this study, hADSCs at passage 2 were used to prepare hADSCs spheroids and hADSCs microspheres. Then, 96-well plates with a U-shaped bottom (EFL-SP101, Engineering for Life of Yongqinquan, Suzhou, China) were utilized for preparing hADSCs spheroids. Initially, an anti-adhesion coating solution (100 μL per well) was added to the 96-well plates without bubbles to create ultralow attachment conditions. Subsequently, the plates were incubated for a minimum of 10 min in a standard humidified incubator (37 °C, 5% CO^2^). After incubation, the coating solution was aspirated and discarded, and the wells were washed three times with PBS. A cell suspension of 10,000 parent or transfected hADSCs in 200 μL of mesenchymal stem cell medium per well was added to the coated ultralow attachment 96-well plates. The plates were placed on the rocking base of a miniaturized wave bioreactor with a controlled rocking angle of 8° and a rocking speed of 20 rpm in a standard humidified incubator. The rocking conditions induced wave motion of the culture medium under defined shear stress, facilitating the spontaneous aggregation of hADSCs. Within 24 h of culture, the cells gradually aggregated, forming hADSCs spheroids (approximately 200 μm in diameter) with ultralow attachment characteristics on the U-shaped bottom wells.

GelMA microspheres were kindly provided by Engineering for Life of Yongqinquan (EFL-MS-C-GM, Suzhou, China) and prepared by optimized emulsion crosslinking technology. The microspheres, with a diameter of approximately 200 μm after swelling, have a high specific surface area. The multimicrosphere assembly scaffold features an interconnected porous structure, providing ample space for cell proliferation to accurately simulate the tissue microenvironment. Prior to the preparation of hADSCs microspheres, GelMA microspheres were sterilized by soaking them in 75% ethanol for 30–60 min. Following sterilization, the microspheres were washed with PBS (1 ×) three times for 15–20 min each time. Subsequently, the sterile GelMA microspheres were transferred to ultralow-adhesion sterile 96-well plates (200 GelMA microspheres per well, approximately 0.4 mg). Mesenchymal stem cell medium was added to the well plates to submerge the GelMA microspheres, which were then allowed to swell fully in a 37 °C incubator for 15–30 min. Next, 200 μL of cell suspension containing a total of 10,000 hADSCs was added to each well. The well plates were also placed on the rocking base of a miniaturized wave bioreactor with a controlled rocking angle of 8° and a rocking speed of 20 rpm in a standard humidified incubator to ensure uniform distribution of hADSCs. Within 24 h of culture, the hADSCs gradually adhered to the surface and proliferated into GelMA microspheres (approximately 200 μm in diameter). The process of changing the medium and collecting culture supernatant should be carried out gently to prevent external forces from causing the cells to detach.

For the collection of hADSCs spheroids-EVs/hADSCs microspheres-EVs, the medium on top of the hADSCs spheroids/hADSCs microspheres was refreshed every 24 h by aspirating 150 μL of culture supernatant and adding 150 μL of fresh mesenchymal stem cell medium containing 5% EVs-free FBS using a 200 μL pipette. The cell culture and culture supernatant collection lasted two consecutive weeks. Live and dead cell staining was performed every three days (i.e., on days 3, 6, 9 and 12) to ensure cell viability. The morphology and size distribution of the hADSCs spheroids/hADSCs microspheres were observed under a confocal laser microscope (Leica TCS SP8 SR, Germany) during the collection of the culture supernatant. Approximately 50 μL of medium per well was retained during medium refreshing to avoid aspirating the hADSCs spheroids/hADSCs microspheres. The culture supernatant was collected, pooled and stored at − 80 °C. Subsequently, EVs were isolated from the culture supernatant through differential ultracentrifugation, as described below. The quality control of EVs was assessed using nanoparticle tracking analysis (NTA) to determine EV concentration and size distribution. Additionally, the morphology of EVs was examined using transmission electron microscopy (TEM). The presence of protein markers CD63, CD81, TSG101, and HSP70 was confirmed through Western blotting.

### EVs purification by differential ultracentrifugation

To obtain EVs-free FBS, a centrifugation process was conducted overnight at a speed of 100000 × g. The hADSCs spheroids or hADSCs microspheres were cultured in 5% EVs-free FBS. Initially, the culture supernatant was centrifuged at 200 × g for 10 min and then at 2000 × g for 15 min to remove cellular debris. The supernatant was collected and centrifuged at 10000 × g for 30 min. Subsequently, the supernatant was ultracentrifuged at 100000 × g for 70 min at 4 °C. The EVs pellet was resuspended in cold sterile PBS and filtered through a 0.22-μm filter. The filtered supernatant was then subjected to ultracentrifugation at 100000 × g for 70 min at 4 °C [21]. The isolated EVs were quantified using a BCA assay.

### Characterization of EVs: particle size/concentration, morphology and protein markers

According to the guidelines of the Minimal Information for Studies of Extracellular Vesicles (MISEV) 2018 (MISEV2018), [22] NTA was utilized to analyze the concentration and size distribution of EVs. Additionally, the morphology was observed under TEM (JEM-1400; JEOL, Tokyo, Japan). Western blotting detected protein markers of CD63, CD81, TSG101, and HSP70.

### The miRNAs sequencing and analysis

`The miRNA sequencing technique was utilized to identify differentially expressed miRNAs in hADSCs spheroids-EVs and hADSCs microspheres-EVs. The NEBNext^®^Multiplex Small RNA Library Prep Set for Illumina^®^ (NEB, USA) was used to construct sequencing libraries, following the manufacturer's instructions and to assign index codes to each sample. Differential expression analysis was conducted using the DESeq R package (3.0.3), with P-values adjusted using the Benjamini & Hochberg method. A corrected P value of 0.05 was set as the threshold for significant differential expression.

### Construction and transfection of plasmid vectors

The CHP + LAMP2B vector encodes the fused gene of CHP and LAMP2B, while the LAMP2B vector only encodes the gene of LAMP2B. The negative control (NC) vector serves as the control. LAMP2B consists of a 29-amino-acid signal peptide, a large N-terminal extramembrane domain, and a C-terminal transmembrane region followed by a very short cytoplasmic tail. The targeting peptide CHP can be fused to the extracellular domain of LAMP2B at the N-terminus. A GNSTM peptide sequence is introduced to protect the targeting peptides from degradation [23]. Oligo sequences encoding the glycosylation motif (GNSTM), chondrocyte-homing peptide (CHP, DWRVIIPPRPSA), and a glycine-serine spacer was synthesized. The final vector was refined to CAP + Lamp2b for simplicity. hADSCs were transfected with these vectors using Lipofectamine 3000 reagent (Invitrogen) according to the manufacturer’s instructions and expanded when the adherent cells reached 50–60% confluence. Stable cells were then seeded in cell plates with a U-shaped bottom to prepare hADSCs spheroids. Three types of EVs (i.e. CHP-EVs, LAMP2B-EVs, and NC-EVs), corresponding to the CHP + LAMP2B vector, LAMP2B vector and NC vector respectively, were collected. The size distribution and morphology of the EVs were examined using NTA and TEM.

### Cellular uptake of EVs

A PKH-26 kit (Sigma‒Aldrich) was used to label EVs according to the manufacturer's instructions. In brief, CHP-EVs and LAMP2B-EVs were incubated with 500 μL of dilution C solution and 4 μL of PKH-26 dye solution for 5 min. The staining process was halted by the addition of 500 μL of 1% bovine serum albumin. After being centrifuged twice at 100000 × g for 70 min, the labeled EVs were obtained and resuspended in 100 μL of cold PBS. The remaining ear chondrocytes were labeled with carboxyfluorescein diacetate succinimidyl ester (CFDA SE) (Beyotime) following the manufacturer's protocol. Briefly, the remaining ear chondrocytes were incubated with a 5 μM working solution of CFDA SE for 10 min. The labeled chondrocytes were washed and centrifuged twice and then resuspended in a culture dish for two-dimensional culture or mixed with the GelMA hydrogel for three-dimensional culture. The labeled EVs were added to the culture dish or mixed with the GelMA hydrogel and the remaining ear chondrocytes for 24 h. Confocal microscopy imaging was used to observe the uptake of EVs.

### Cell cycle assay

A Cell Cycle Analysis Kit (Beyotime) was used to detect the cell cycle distribution of the remaining ear chondrocytes. The remaining ear chondrocytes (5 × 10^5) were cultured on 6-well plates and incubated overnight at 37 °C. The cells were then treated for 72 h, followed by harvesting and washing with cold PBS. Cell fixation was achieved by adding 1 mL of cold 70% ethanol. The cells were subsequently resuspended in binding buffer and stained with propidium iodide (PI) according to the manufacturer's instructions. Flow cytometric analysis was used to detect and quantify the control and treated cells.

### Cell apoptosis assay

The remaining ear chondrocytes were cultured overnight in 6-well plates at 37 °C. The cells were then subjected to different treatments for 72 h. After being harvested and washed with cold PBS, the cells were stained with Annexin V and propidium iodide (PI), according to the manufacturer's protocol. Flow cytometric analysis was used to detect and quantify the control and treated cells.

### CCK8 assay

The proliferation of remaining ear chondrocytes was assessed using the CCK-8 assay (Dojindo, Japan) according to the manufacturer's instructions. Cell proliferation was determined by measuring the absorbance at 450 nm at 0, 24, 48, 72, and 96 h after treatment.

### Western blot

In brief, the proteins were denatured and then separated on polyacrylamide gels with a concentration range of 4%-12%. The separated proteins were then electroblotted onto a membrane and subsequently probed with a primary antibody. After that, the membrane was incubated with a secondary antibody coupled with HRP against the primary antibody. To visualize the protein bands, the membrane was incubated with SuperSignal West Pico Chemiluminescent Substrate (Thermo Fisher Scientific). The specific primary and secondary antibodies used are listed in Additional file 1.

### Real‐time quantitative PCR (RT-qPCR)

RNA was extracted from chondrocytes/tissue-engineered cartilage samples using TRIzolTM (Invitrogen). Reverse transcription of the RNA was carried out with the PrimeScriptTM RT reagent kit (Takala). RT-qPCR was performed in 20 μL reaction volumes using SYBR Green I Master Mix (Roche) following the manufacturer's instructions. GAPDH served as the internal reference for mRNA. The relative gene expression was determined using the 2^−ΔΔCT^ method. The primers used are listed in Additional file 2**.**

### Live & Dead cell viability assay

The working solution of calcein AM at a concentration of 2 μM and PI at a concentration of 4.5 μM was prepared initially. The samples were then incubated with this solution at room temperature for a duration of 15 min. Cell viability was subsequently observed and quantified using a confocal laser microscope.

### Sulfated glycosaminoglycan (s-GAG) and DNA quantification

The samples were diced and then enzymatically digested with proteinase K (Solarbio) at 65 °C for 5 h. The resulting test materials were mixed with 1000 μL of Blyscan dye reagent and gently shaken for 30 min. The dye concentrations in both the samples and standards were measured at 656 nm. The readings were normalized to the DNA content (s-GAG/DNA).

### Histology and immunohistochemistry

To examine s-GAG deposition, tissue-engineered cartilage samples were cultured in vitro and in vivo. The samples were then fixed, dehydrated, and embedded in paraffin. Standard protocols were followed to cut serial sections and stain the slides with H&E, Safranin-O, Alcian blue, and Toluidine blue. Collagen II, Collagen I, Aggrecan, SOX9, and COMP were detected via immunohistochemistry using a two-step immunohistochemical detection kit (BOSTER). The Average Optical Density (AOD) was calculated to semiquantitatively evaluate the deposition of cartilage extracellular matrix and proteins expression using Image Pro Plus 6.0 software (Media Cybernetics, Rockville, MD, USA). The antibodies used for immunohistochemistry are listed in Additional file 1.

### In vitro* two-dimensional culture of remaining ear chondrocytes in an M1 macrophage-infiltrated microenvironment*

The Transwell coculture system was employed to simulate an environment infiltrated by M1 macrophages. In this system, the lower chamber was seeded with ear chondrocytes, while the upper chamber contained an equal number of M1 macrophages. The remaining ear chondrocytes were divided into three groups: the CHP-EVs-treated group, LAMP2B-EVs-treated group, and Blank group (Untreated group). EVs at a concentration of 10 μg/mL were used to treat the remaining ear chondrocytes while the Blank group received an equivalent volume of PBS.

### In vitro* three-dimensional culture of tissue‐engineered cartilage hydrogels in an M1 macrophage-infiltrated microenvironment*

The porous GelMA hydrogel was lyophilized and then dissolved in PBS with a 10% w/v concentration. The chondrocytes and EVs were resuspended in the GelMA hydrogel to prepare a bioink with a cell density of approximately 1 × 10^7^/mL. The bioink was poured onto a round mold and cured using a 405-nm blue light source for 15 s to prepare tissue-engineered cartilage hydrogels. The remaining ear chondrocytes were divided into three groups: the CHP-EVs-treated group, LAMP2B-EVs-treated group, and Blank group. The concentration of EVs was 0.5 μg/μL. The tissue-engineered cartilage samples were cocultured with M1 macrophage using a Transwell coculture system to mimic a microenvironment infiltrated by M1 macrophage. Tissue-engineered cartilage samples were seeded in the lower chamber, while an equal number of M1 macrophage were seeded in the upper chamber. The tissue-engineered cartilage hydrogel samples were soaked in DMEM for 2 weeks, and the medium was changed every 2 days.

### Animal experiment

The animal experiment in this study was approved by the Ethics Committee of Animal Care and Experiment at the Plastic Surgery Hospital and Institute of the Chinese Academy of Medical Sciences & Peking Union Medical College [2023 (77)]. The surgical procedures followed the guidelines for the care and use of laboratory animals. Eighteen female BALB/c nude mice, aged 6 weeks, were randomly divided into three groups: (1) the CHP-EVs-treated group, (2) the LAMP2B-EVs-treated group, and (3) the Blank group. The density of the final remaining ear chondrocytes was approximately 1 × 10^7^/mL. The concentration of EVs used was 0.5 μg/μL. The bioink was poured onto a round mold and cured with a 405-nm blue light source for 15 s to prepare tissue-engineered cartilage samples. A median longitudinal incision was designed on the back, with an approximately 0.8 cm × 0.6 cm subcutaneous pocket for implanting the freshly prepared tissue-engineered cartilage samples. The same number of freshly-induced M1 macrophage was injected adjacent to the tissue-engineered cartilage samples every 3 days. The animals were sacrificed 4 weeks after surgery, and the tissue-engineered cartilage samples were collected for analysis.

### Statistical analysis

Statistical analysis was carried out using GraphPad Prism version 8.0 (GraphPad, San Diego, CA, USA) software. The two-tailed Student’s t-test was used for comparing two groups. One-way or two-way analysis of variance (ANOVA) followed by Tukey’s post hoc test was conducted for multiple comparisons. Difference was considered statistically significant when p < 0.05.

## Results

### Identification of hADSCs and three-dimensional cell culture-derived hADSCs-EVs

**Fig. 1:**
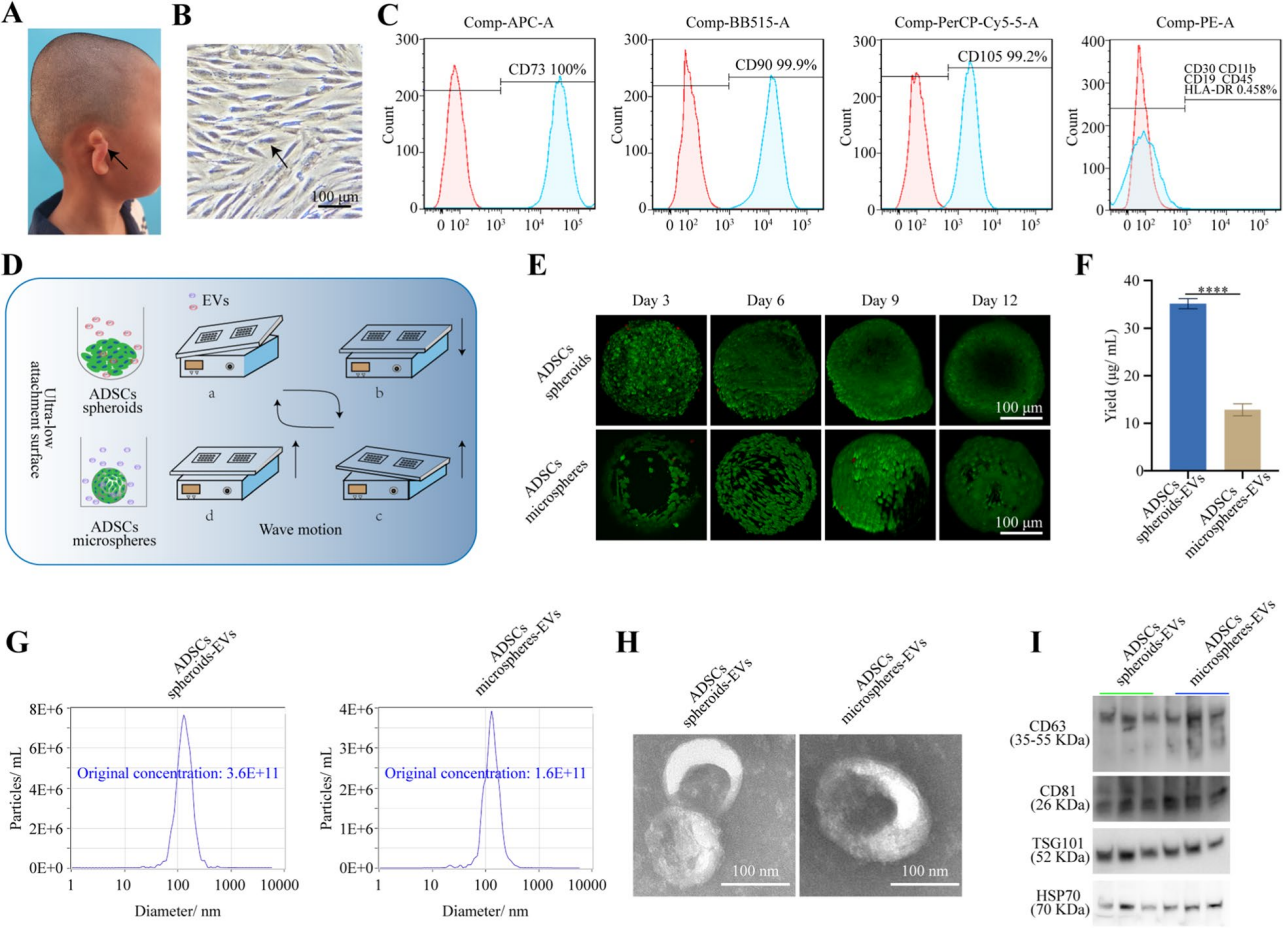
Characteristics of hADSCs and hADSCs-EVs. **A** Representative image of microtia children. **B** Representative image of hADSCs under microscopy. Scale bar: 100 μm. **C** Flow cytometry analysis for the identification of hADSCs. **D** Diagram of producing hADSCs spheroids-EVs and hADSCs microspheres-EVs using a miniaturized wave bioreactor. **E** Live & Dead staining assay. Green: live cells. Red: dead cells. Scale bar: 100 μm. **F** Comparison of EVs production. **G** NTA detection. (H) TEM detection. Scale bar: 100 nm. **I** Western blotting assay. The data are presented as the mean ± SD

The remaining ear chondrocytes were successfully isolated from the remaining ear cartilage of microtia children (Fig. 1A). Microscopy revealed that hADSCs exhibited a spindle-shaped appearance (Fig. 1B). Flow cytometry analysis detected the surface markers of hADSCs, including CD73, CD90, and CD105 (Fig. 1C), which met the criteria for identifying MSCs. Two three-dimensional culture methods were utilized for isolating hADSCs-EVs. hADSCs spheroids were prepared to secrete hADSCs spheroids-EVs while GelMA microspheres were utilized to secrete hADSCs microspheres-EVs. Both the hADSCs spheroids and hADSCs microspheres were cultured under wave motion in a miniaturized wave bioreactor (Fig. 1D). Both hADSCs spheroids and hADSCs microspheres exhibited high cell viability during the process of EVs harvesting, as confirmed by Live & Dead cell viability assay conducted every three days (Fig. 1E). To enable long-term three-dimensional culture, a harvesting method pipeline was developed. This method involved separating the conditioned medium for EVs isolation and subsequently resuspended hADSCs spheroids and hADSCs microspheres every 24 h. This step of separating the conditioned medium did not damage the hADSCs spheroids and hADSCs microspheres, allowing for the collection of the EVs-rich conditioned medium for further isolation. The remaining pellet (hADSCs spheroids and hADSCs microspheres) was seeded in the well plates for further culture by gently suspending it in fresh medium. The hADSCs spheroids secreted more EVs than hADSCs microspheres (Fig. 1F). NTA showed that approximately 98.5% of the hADSCs spheroids-derived EVs had diameters between 30 and 150 nm and an original concentration of 3.6^11 particles/mL. Approximately 98.7% of the hADSCs microsphere-EVs had diameters between 30 and 150 nm and an original concentration of 1.6^11 particles/mL (Fig. 1G). Both hADSCs spheroids-EVs and hADSCs microspheres-EVs exhibited cup-shaped vesicles, as observed under TEM (Fig. 1H). Furthermore, Western blotting revealed the expression of CD63, CD81, TSG101, and HSP70 on the surface of hADSCs spheroids-EVs and hADSCs microspheres-EVs (Fig. 1I). Overall, these findings confirm the successful isolation of hADSCs spheroids-EVs and hADSCs microspheres-EVs. The production pipeline of hADSCs spheroids or hADSCs microspheres-based EVs using a harvesting method had no effect on the size distribution, morphology and protein markers. Compared with hADSCs microspheres, hADSCs spheroids were more effective at enhancing EVs production.

### Comparison of miRNA profiles between hADSCs spheroids-EVs and hADSCs microspheres-EVs

**Fig. 2:**
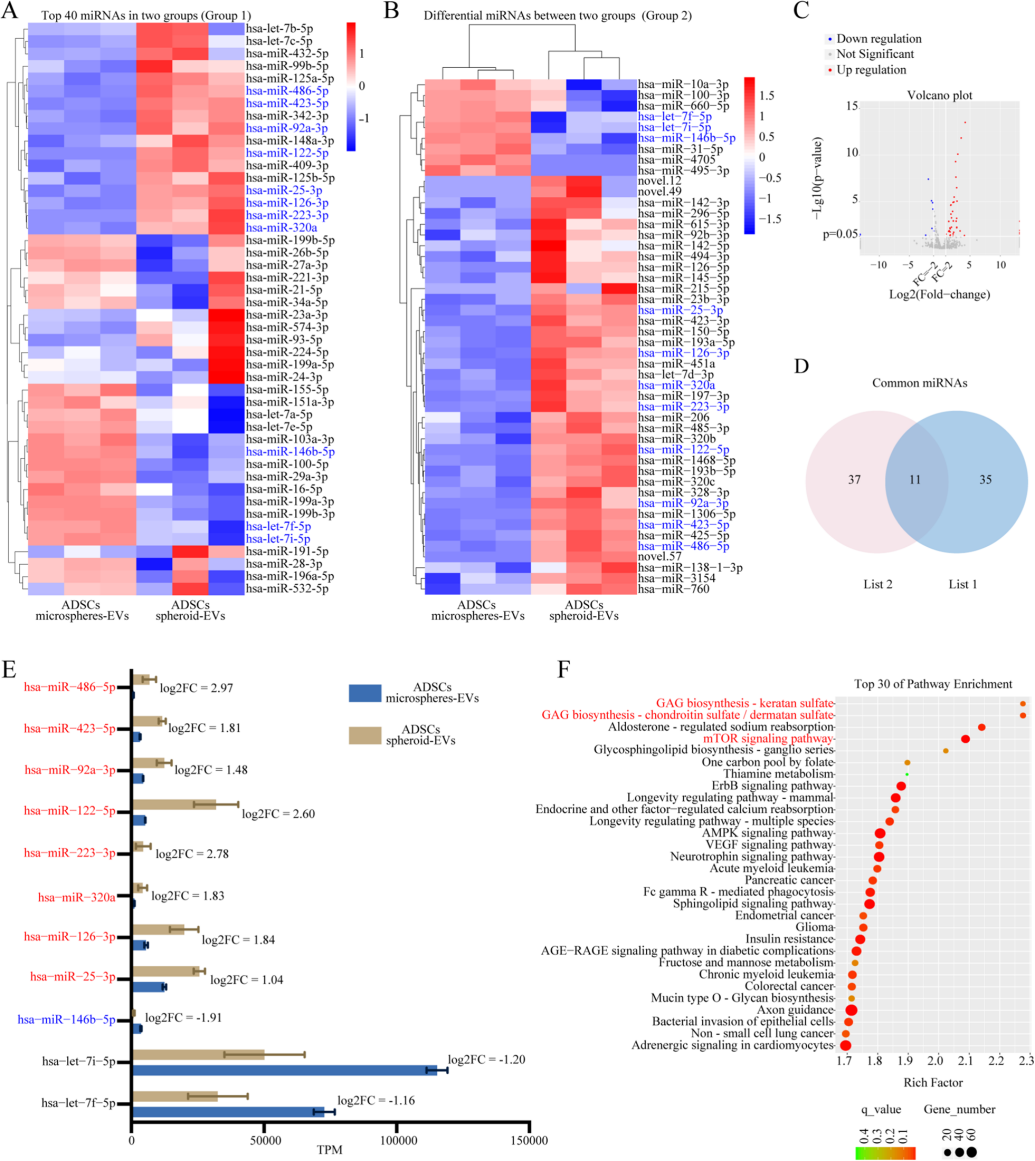
The miRNA sequencing of hADSCs spheroids-EVs and hADSCs microspheres-EVs. **A** The 40 most highly expressed miRNAs (Group 1). **B** The differentially expressed miRNAs (Group 2). **C** Volcano plot showing the number of upregulated and downregulated miRNAs. **D** The common miRNAs between group 1 and group 2. **E** The specific details of the common miRNAs. **F** KEGG enrichment analysis of the common miRNAs

EVs are predominantly affected by various physiological conditions, suggesting that changes in the culture microenvironment of secreting cells can induce alterations in the miRNA profiles of EVs. MiRNAs, which are crucial components of EVs, play significant roles in regulating gene expression and biological function by binding to the 3ʹUTRs of target genes. Fig. 2A presented the 40 most highly expressed miRNAs within hADSCs spheroids-EVs and hADSCs microspheres-EVs (Group 1). Fig. 2B showed a total of 48 differentially expressed miRNAs identified between hADSCs spheroids-EVs and hADSCs microspheres-EVs (Group 2), with 39 upregulated miRNAs and 9 downregulated miRNAs in hADSCs spheroids-EVs (Fig. 2C). As shown in Fig. 2D, among the 40 most highly expressed miRNAs, there were 11 differentially expressed miRNAs (Fig. 2E). The upregulation of eight miRNAs (hsa-miR-486-5p, hsa-miR-423-5p, hsa-miR-92a-3p, hsa-miR-122-5p, hsa-miR-223-3p, hsa-miR-320a, hsa-miR-126-3p, and hsa-miR-25-3p) and the downregulation of hsa-miR-146b-5p within hADSCs spheroids-EVs were reported to improve chondrocyte fate and enhance chondrogenesis. No studies have reported the roles of hsa-let-7i-5p and hsa-let-7f-5p in cartilage formation. The roles of the 11 differentially expressed miRNAs in cartilage regeneration were summarized in Table 1. Subsequently, KEGG enrichment analysis was conducted to analyze the differentially regulated genes related to the 11 differentially expressed miRNAs mentioned above, and the top 30 pathways were identified. Among these pathways, some were enriched in GAG biosynthesis, specifically keratan sulfate and chondroitin sulfate/dermatan sulfate, and mTOR signaling pathway, all of which are involved in chondrogenesis (Fig. 2F). In summary, hADSCs spheroids-EVs exhibited greater levels of chondrogenesis-related miRNAs compared with hADSCs microspheres-EVs. Therefore, we selected an innovative pipeline based on hADSCs spheroids to obtain EVs with excellent chondrogenesis potential.

**Table 1:** The roles of the 11 differentially expressed miRNAs in cartilage regeneration

miRNA	Regulation level	Roles in cartilage formation	References
miR-486-5p	Up	Inhibiting endoplasmic reticulum stress, alleviating chondrocytes apoptosis, promoting matrix regeneration, and regulating macrophage polarization	Wang et al. [43], Li et al. [44]
miR-423-5p	Up	Inhibiting chondrocytes inflammation	Chen et al. [45]
miR-92a-3p	Up	Exhibiting pro-chondrogenic and chondrocyte protective effects by targeting SMAD6/7; Promoting proliferation and chondrogenesis of chondrocytes and MSCs by targeting WNT5A; Regulating cartilage development and homeostasis by targeting HDAC2; Downregulating the expression of ADAMTS-4/5	Zheng et al. [46]; Mao et al. [47], Mao et al. [48], Mao et al. [49]
miR-122-5p	Up	Promoting chondrogenic differentiation of MSCs by activating Wnt1/β-catenin pathway	Alahdal et al. [50]
miR-223-3p	Up	Inhibiting apoptosis and inflammatory response in chondrocytes by targeting NLRP3	Dong et al. [51], Mao et al. [52]
miR-320a	Up	Promoting chondrocytes proliferation, reducing apoptosis and inflammation by targeting DAZAP1 and MAPK pathways; Alleviating cartilage degradation	Mao et al. [52], Peng et al. [53]
miR-126-3p	Up	Enhancing chondrocytes viability, reducing apoptosis and inflammation, and promoting chondrogenesis	Li et al. [54], Zhou et al. [55]
miR-25-3p	Up	Alleviating chondrocytes pyroptosis by inhibiting CPEB1; Promoting proliferation and inhibiting apoptosis by targeting IGFBP7; Enhancing cell viability and suppressing apoptosis by inhibiting JPX	Wang et al. [56], He et al. [57], Ren et al. [58]
miR-146b-5p	Down	Controlling inflammatory response and promoting chondrogenesis	Jia et al. [59]
let-7i-5p	Down	No reporting	–
let-7f-5p	Down	No reporting	–

### Engineering hADSCs spheroids-EVs with chondrocyte-targeting potential

Figure 3A showed the construction of CHP + LAMP2B plasmid vector, the transfection of hADSCs and the production of CHP-Exo. Fig. 3B demonstrated successful vector transfection into hADSCs. Western blotting of hADSCs spheroids revealed significantly increased levels of LAMP2B in hADSCs spheroids after transfection with the LAMP2B or CHP + LAMP2B vector. All the transfected hADSCs exhibited similar expression levels of CD81, CD63, HSP70, and TSG101 (Fig. 3C**&D**). NTA revealed that the purified EVs had an average diameter of approximately 100 nm (Fig. 3E). TEM analysis of the purified EVs revealed typical cup-shaped structures (Fig. 3F). The shape and size distribution of the engineered hADSCs spheroids-EVs (CHP-EVs) were similar to those of the LAMP2B-EVs, NC-EVs and PBS-EVs, indicating that the insertion of CHP had no effect on the shape or size distribution of the EVs.

**Figure:**
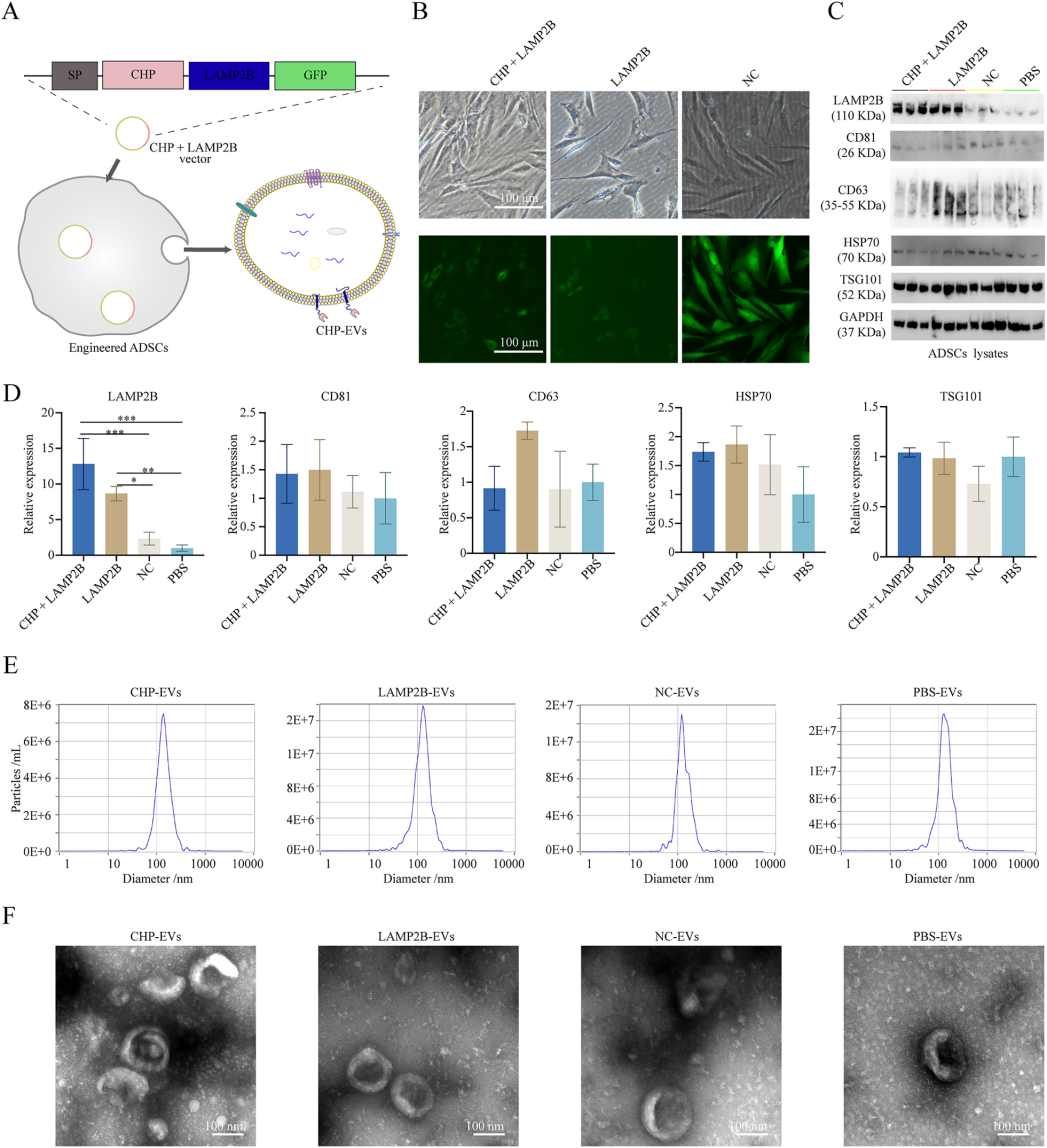
Fig. 3 Engineering hADSCs spheroids-EVs with CHP (CHP-EVs). **A** Diagram of CHP-EVs design. **B** Successful transfection of three vectors into hADSCs. Scale bar: 100 μm. **C** Western blotting detection. **D** Semiquantitative analysis of protein expression. **E** NTA detection. **F** TEM detection. Scale bar: 100 nm. The data are presented as the mean ± SD

### Two-dimensional and three-dimensional intracellular uptake of CHP-EVs in a microenvironment infiltrated by M1 macrophage

**Fig. 4:**
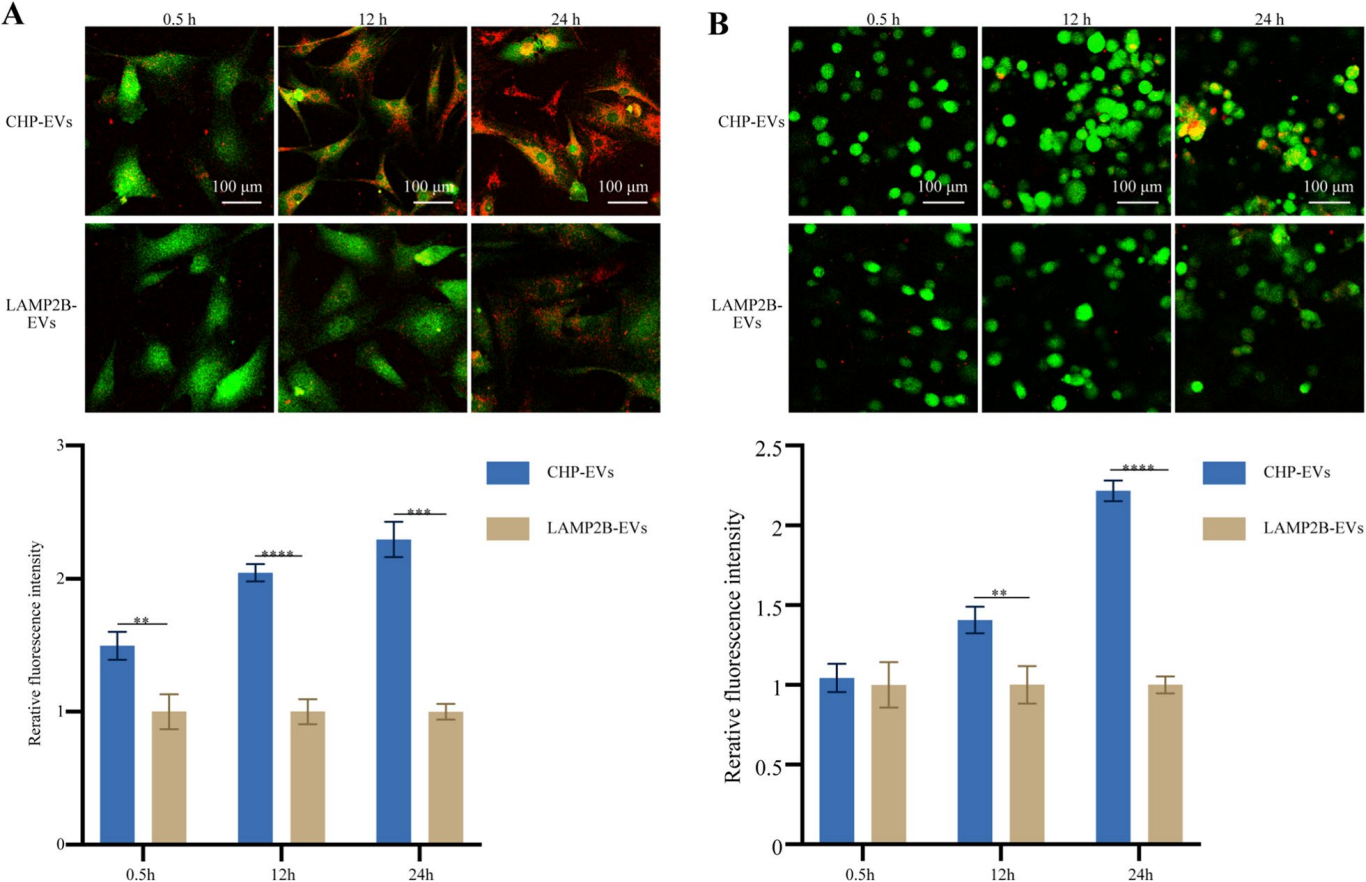
Cellular uptake of EVs. **A** In vitro two-dimensional uptake of EVs. **B** In vitro three-dimensional uptake of EVs. The merged images of CFDA SE-labeled remaining ear chondrocytes (green) and PKH-26-labeled EVs (red) at 0.5 h, 12 h and 24 h showed the presence of EVs in the cytoplasm. Scale bar: 100 μm. The data are presented as the mean ± SD

When we incubated the PKH26-labeled CHP-EVs with CFSE-labeled remaining ear chondrocytes, we observed fluorescence signals inside the chondrocytes, indicating successful uptake of the EVs by the cells. The cells treated with CHP-EVs showed significantly greater signals than did the cells treated with LAMP2B-EVs without surface-functionalized CHP sequences at the two-dimensional culture level (Fig. 4A) and three-dimensional culture level (Fig. 4B). These results suggest that the CHP plays a crucial role in facilitating the rapid uptake of EVs by remaining ear chondrocytes, with exceptional tissue penetration capability through densely packed hydrogel scaffolds and specific delivery of therapeutic miRNAs to the targeted sites.

### CHP-EVs improved remaining ear chondrocytes fate by enhancing cell proliferation, regulating cell cycle, reducing cell apoptosis, and promoting cartilage formation in vitro in the M1 macrophage-infiltrated microenvironment

**Fig. 5:**
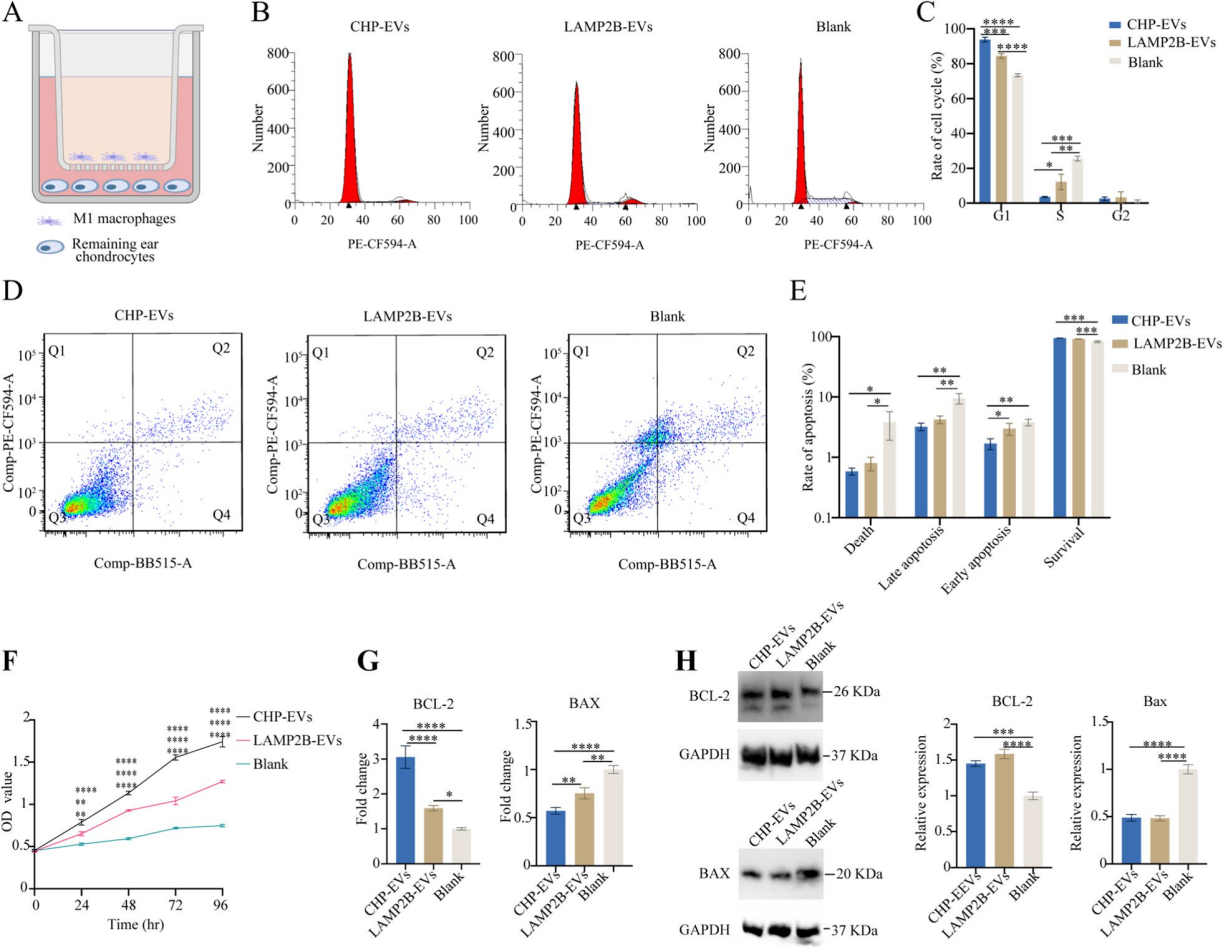
The effects of CHP-EVs on the fate of remaining ear chondrocytes. **A** Illustration of the M1 macrophage-infiltrated microenvironment model. **B** Representative image of cell cycle assay. **C** Quantitative analysis of cell cycle assay. **D** Representative image of cell apoptosis assay **E** Quantitative analysis of cell apoptosis assay. **F** CCK-8 assay. Upper “*”: CHP-EVs compared to Blank. Middle “*”: LAMP2B-EVs compared to Blank. Lower “*”: CHP-EVs compared to LAMP2B-EVs. **G** Analysis of cell apoptosis-associated genes by RT-qPCR. **H** Western blotting assay and semiquantitative analysis. The data are presented as the mean ± SD

The remaining ear chondrocytes were cocultured with M1 macrophage using a Transwell system in vitro (Fig. 5A). To verify the ability of CHP-EVs to improve the fate of remaining ear chondrocytes, we performed various cell function assays. Flow cytometry analysis showed that both the CHP-EVs-treated group and LAMP2B-EVs-treated group exhibited a significant decrease in the percentage of S-phase cells and a significant increase in the percentage of G1-phase cells compared to the Blank group. Notably, the CHP-EVs-treated group outperformed the LAMP2B-EVs-treated group in decreasing the percentage of S-phase cells and increasing the percentage of G1-phase cells, indicating that CHP-EVs inhibited S-phase cell cycle arrest in the remaining ear chondrocytes (Fig. 5B&C). Flow cytometry analysis revealed that both the CHP-EVs-treated group and the LAMP2B-EVs-treated group demonstrated a significant decrease in the percentage of cell death, late apoptosis and early apoptosis but a significant increase in the percentage of cell survival compared to the Blank group. Furthermore, the CHP-EVs-treated group outperformed the LAMP2B-EVs-treated group in decreasing the percentage of early apoptosis (Fig. 5D&E). The CCK-8 assay demonstrated that both the CHP-EVs-treated group and LAMP2B-EVs-treated group exhibited significantly enhanced cell proliferation compared to the Blank group. However, CHP-EVs treatment had a major impact on promoting the proliferation of remaining ear chondrocytes (Fig. 5F). RT-qPCR revealed that the CHP-EVs treatment had the greatest effect on increasing the gene expression of BCL-2 and decreasing the gene expression of BAX (Fig. 5G). Western blotting results indicated that CHP-EVs-treated group and LAMP2B-EVs-treated group were similar to increase protein levels of BCL-2 and decrease protein levels of BAX (Fig. 5H).

**Fig. 6:**
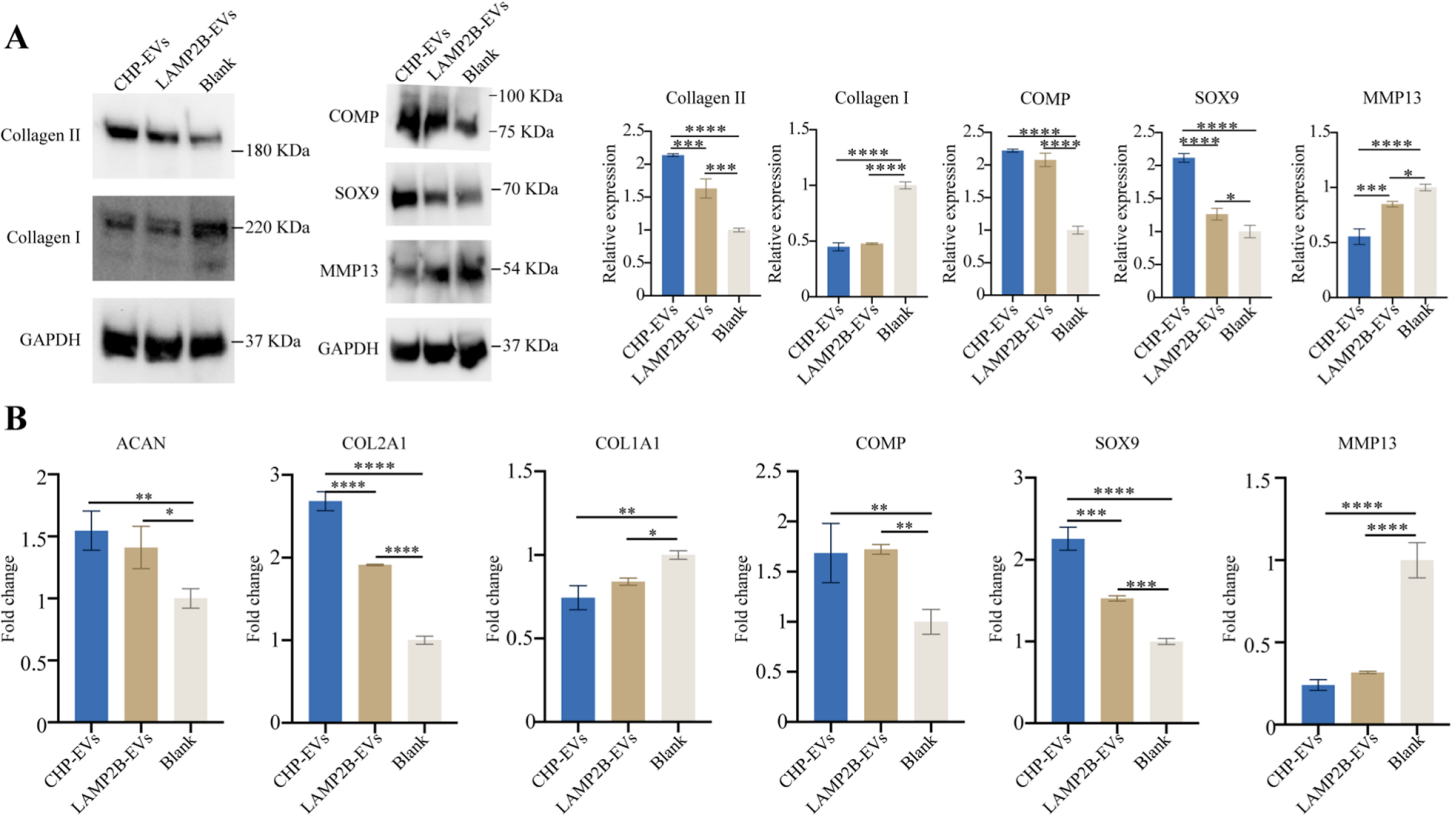
The effects of CHP-EVs on the chondrogenesis potential. **A** Western blotting assay. **B** RT-qPCR assay. The data are presented as the mean ± SD

Given the propensity of remaining ear chondrocytes proliferation to induce dedifferentiation and subsequent loss of chondrocytic phenotype, we aimed to investigate whether the induction of chondrocytes proliferation through CHP-EVs would affect the synthesis of cartilage matrix. Fig. 6A showed that both the CHP-EVs treated group and LAMP2B-EVs treated group significantly increased level of chondrogenesis-associated proteins, such as Collagen II, COMP, and SOX9, while reducing the expression of Collagen I and MMP13. Notably, the CHP-EVs treated group had the most excellent effect on the protein expression of Collagen II, SOX9, and MMP13. RT-qPCR revealed that the CHP-EVs treated group and LAMP2B-EVs treated group exhibited significantly increased the gene expression of ACAN, COL2A1, COMP, and SOX9, but decreased gene expression of COL1A1 and MMP13. In particular, CHP-EVs treatment had the most excellent effect on the gene expression of COL2A1 and SOX9. (Fig. 6B) Overall, these findings demonstrated that the CHP-EVs treatment was more effective at promoting cell proliferation, inhibiting cell apoptosis, and enhancing cartilage formation.

### *The effects of CHP-EVs on tissue-engineered cartilage hydrogels *in vitro* in the M1 macrophage-infiltrated microenvironment*

**Fig. 7:**
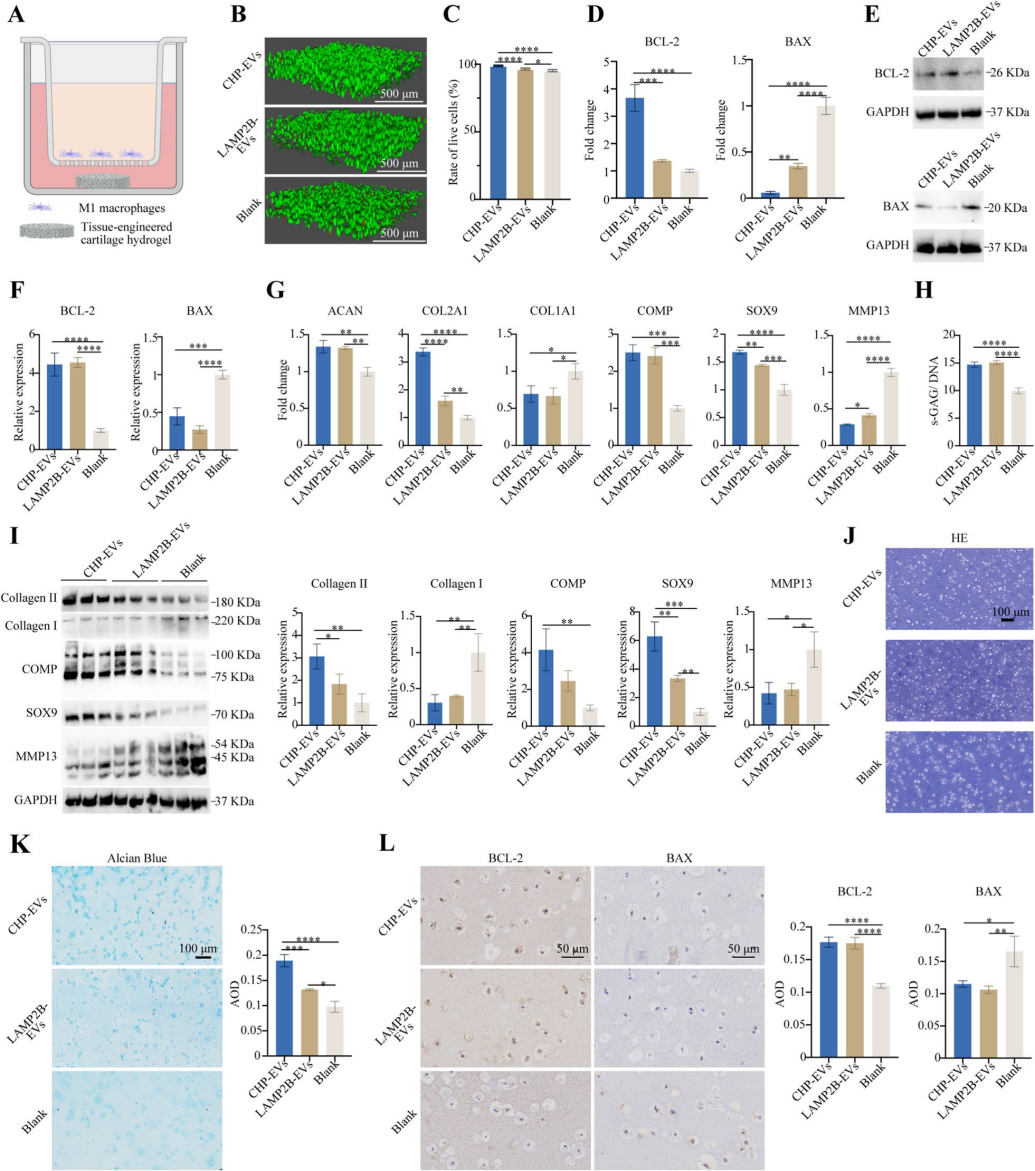
The effects of CHP-EVs on tissue-engineered cartilage hydrogels. **A** Illustration of a M1 macrophage-infiltrated microenvironment. **B** Representative image of the Live/Dead staining assay. Green: live cells. Red: dead cells. Scale bar: 100 μm. **C** Quantitative analysis of the Live/Dead staining assay. **D** RT-qPCR assay. **E** Western blotting assay. **F** Semiquantitative analysis of cell apoptosis-associated proteins. **G** Analysis of chondrogenesis-associated genes by RT-qPCR assay. **H** Quantitative analysis of standardized s-GAG. **I** Analysis of chondrogenesis-associated proteins by Western blotting assay. **J** H&E staining. Scale bars: 100 μm. **K** Alcian blue staining and semiquantitative analysis. Scale bars: 100 μm. **L** Immunohistochemical staining and semiquantitative analysis. Scale bars: 100 μm. The data are presented as the mean ± SD

**Fig. 8:**
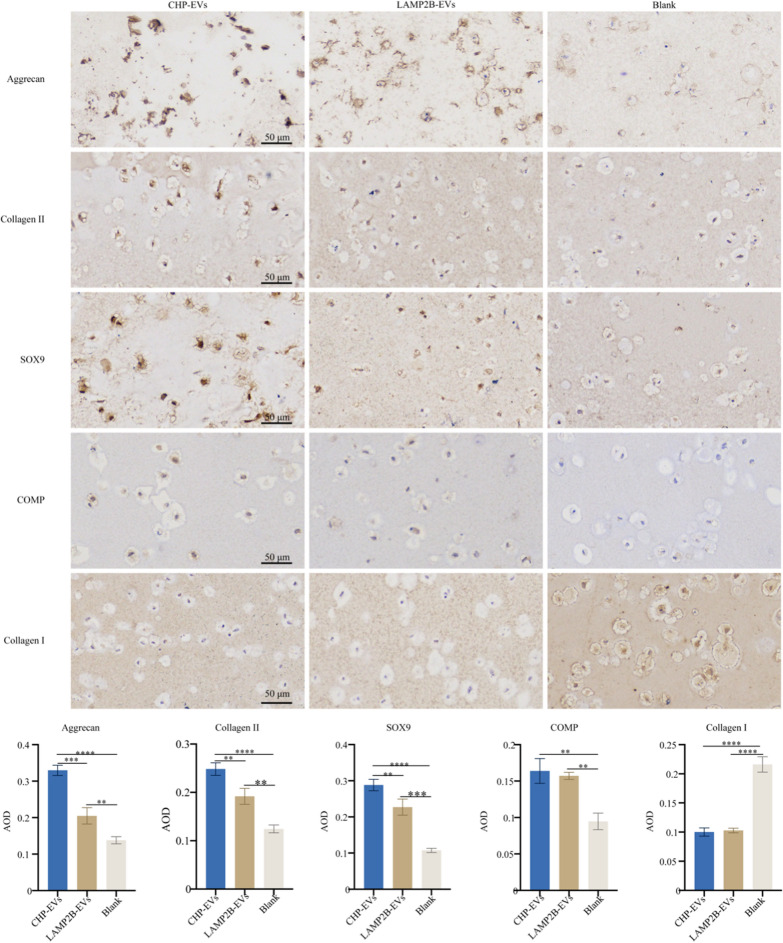
Immunohistochemical staining and semiquantitative analysis of chondrogenesis-associated proteins. Scale bars: 50 μm. The data are presented as the mean ± SD

The tissue-engineered cartilage hydrogels were cocultured with M1 macrophage using the Transwell system for two weeks (Fig. 7A). The Living & Dead staining results demonstrated that CHP-EVs were more effective in increasing the rate of cell survival than the other two treatments (Fig. 7B&C). Compared with the other two groups, the CHP-EVs-treated group significantly increased the gene expression of BCL-2 while decreased the gene expression of BAX (Fig. 7D). There was no significant difference between the CHP-EVs-treated group and LAMP2B-EVs-treated group in regulating the protein expression of BCL-2 and BAX (Fig. 7E&F). Both the CHP-EVs-treated group and LAMP2B-EVs-treated group upregulated the gene expression of ACAN, COL2A1, COMP, and SOX9, but downregulated the gene expression of COL1A1 and MMP13. Specifically, the CHP-EVs treatment had the most excellent impact on the gene expression of COL2A1, SOX9, and MMP13 (Fig. 7G). No difference was seen between the CHP-EVs-treated group and LAMP2B-EVs-treated group in increasing the synthesis of cartilaginous matrix (Glycosaminoglycan, GAG) (Fig. 7H). Furthermore, both the CHP-EVs-treated group and the LAMP2B-EVs-treated group significantly upregulated the protein expression of Collagen II, COMP, and SOX9, and downregulated the level of Collagen I and MMP13. Specifically, the CHP-EVs treatment had a major effect on Collagen II and SOX9 (Fig. 7I). H&E staining showed the cartilage lacuna-like structures (Fig. 7J). Additionally, the secretion of cartilaginous matrix was significantly stained with Alcian blue in the CHP-EVs treated group, compared to the other two groups (Fig. 7K). Immunohistochemical staining revealed that both the CHP-EVs-treated group and LAMP2B-EVs-treated group significantly increased the protein level of BCL-2 and decreased the level of BAX, compared to the Blank group (Fig. 7L). Moreover, both the CHP-EVs-treated group and LAMP2B-EVs-treated group significantly upregulated the protein level of Aggrecan, Collagen II, SOX9, and COMP and downregulated the level of Collagen I. Notably, the CHP-EVs-treated group exhibited the most excellent effect on Aggrecan, Collagen II, and SOX9. (Fig. 8).

### *Histological evaluation of cartilage formation potential of CHP-EVs *in vivo* in an M1 macrophage-infiltrated microenvironment*

**Fig. 9:**
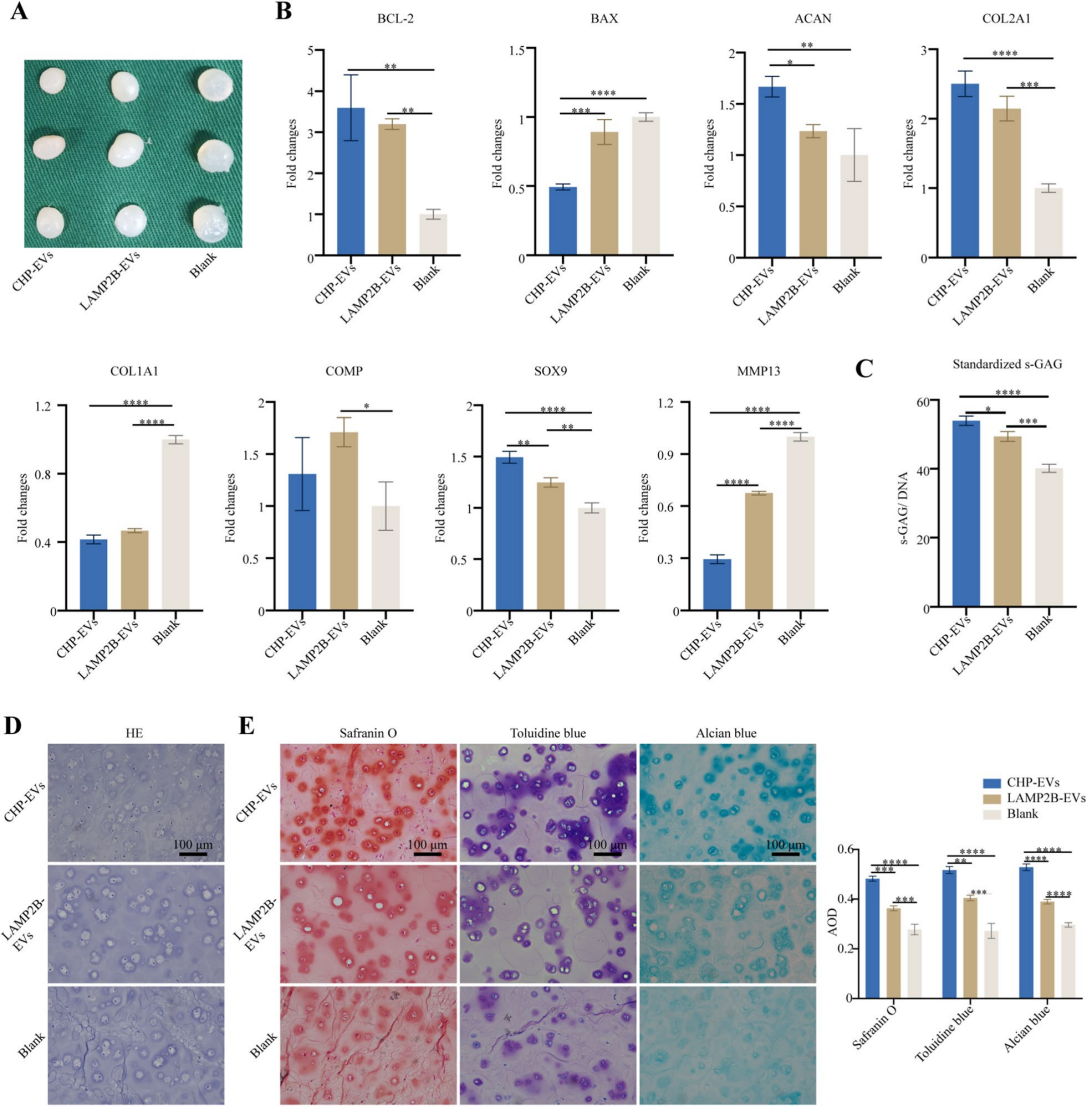
In vivo effects of CHP-EVs on tissue-engineered cartilage hydrogels. **A** Gross observation. **B** Analysis of cell apoptosis and chondrogenesis-associated genes. **C** Quantitative analysis of standardized s-GAG. **D** Histological evaluation of tissue-engineered cartilage using HE staining. Scale bars: 100 μm. **E** Histological evaluation of tissue-engineered cartilage using Safranin O, Toluidine blue, and Alcian blue staining and their semiquantitative analysis. Scale bars: 100 μm. The data are presented as the mean ± SD

**Fig. 10:**
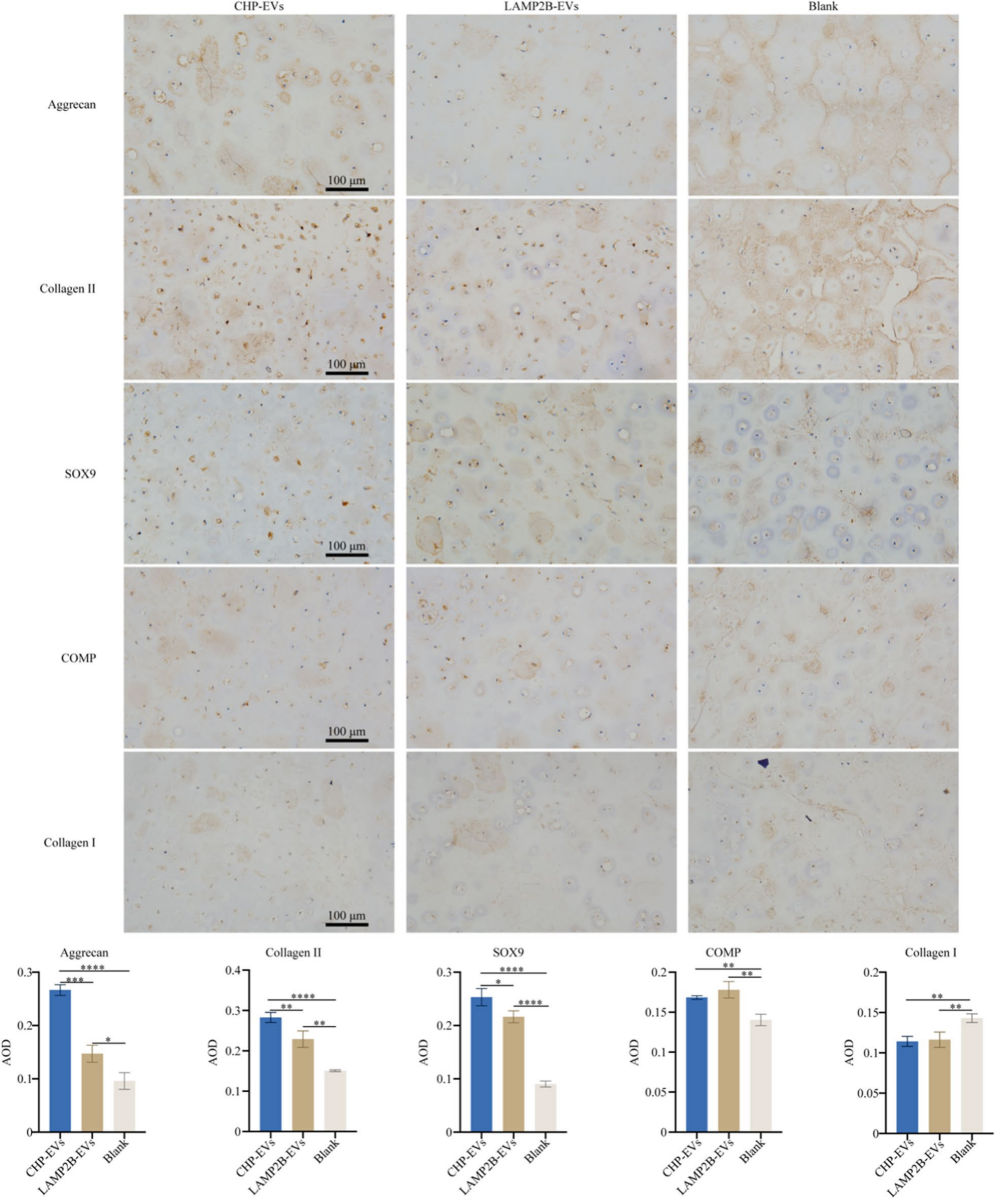
Immunohistochemical staining and semiquantitative analysis of chondrogenesis-associated proteins. Scale bars: 100 μm. The data are presented as the mean ± SD

The results showed that the tissue-engineered cartilage samples in the CHP-EV-treated group exhibited a more compact and ivory-white appearance, with a tough and elastic texture (Fig. 9A). RT-qPCR revealed that both the CHP-EVs-treated group and the LAMP2B-EVs-treated group significantly increased the gene expression of BCL-2, while only the CHP-EVs-treated group significantly decreased the gene expression of BAX, compared to the Blank group. Furthermore, both the CHP-EVs-treated group and the LAMP2B-EVs-treated group showed significant upregulation of COL2A1 and SOX9, and significant downregulation of COL1A1 and MMP13, compared to the blank group. In particular, compared to the other two group, the CHP-EVs-treated group had a major impact on the gene expression of BAX, ACAN, SOX9 and MMP13. Notably, only the LAMP2B-EVs-treated group significantly increased the gene expression of COMP. (Fig. 9B) After 4 weeks of CHP-EVs treatment, the levels of s-GAG/DNA were significantly greater than those in the other two groups, suggesting that CHP-EVs had a major impact on enhancing the biosynthesis of s-GAG (Fig. 9C). H&E staining revealed the cartilage lacuna-like structures in all groups (Fig. 9D). Furthermore, the CHP-EVs-treated group show the most excellent synthesis of cartilaginous matrix, as confirmed by Safranin O, Toluidine blue, and Alcian blue staining, which was consistent with the results of s-GAG quantification analysis (Fig. 9E). Immunohistochemical staining revealed that the CHP-EVs-treated group and LAMP2B-EVs-treated group significantly upregulated the protein level of Aggrecan, Collagen II, SOX9, and COMP and downregulated the level of Collagen I. Notably, the CHP-EVs-treated group exhibited the most excellent effect on the regulation of Aggrecan, Collagen II, and SOX9. (Fig. 10).

## Discussion

Tissue-engineered ears present a promising solution for reconstructing external ears, but their practical application in clinical settings has not yet become widespread. At our ear reconstruction center, we successfully prepared patient-specific ear-shaped cartilage [2]. Although we initially achieved satisfactory aesthetic results within the first three years, long-term follow-up revealed a gradual loss of shape and eventual collapse of the engineered ears. This can partially be attributed to the infiltration of M1 macrophage in the microenvironment surrounding the tissue-engineered ear.

M1 macrophages, activated by interferon-γ (IFN-γ), lipopolysaccharide (LPS), or tumor necrosis factor alpha (TNF-α), secrete proinflammatory cytokines and mediators such as TNF-α, interleukin (IL)-1, IL-6, and IL-12 [24]. Studies have shown that M1 macrophage infiltration inhibits cartilage regeneration by promoting cartilage degeneration and hypertrophic chondrocyte differentiation through various mechanisms. For example, M1 macrophage polarization, characterized by increased expression of IL-1, IL-6, and TNF-α, worsens cartilage degeneration and promotes hypertrophic chondrocyte differentiation through the secretion of Rspo2 and activation of β-catenin signaling in chondrocytes [6]. The inhibition of M1 activation and repolarization to the M2 phenotype have anti-inflammatory effects to support chondrogenesis [7, 25, 26]. Inflammatory M1 macrophage-like cytokines play a crucial role in the destructive processes observed in cartilage tissue, primarily by upregulating metalloproteinases (MMP3, MMP9, and MMP13) [27]. Recently, EVs released from infiltrated M1 macrophage have been shown to induce noncanonical pyroptosis in chondrocytes and promote cartilage catabolism by activating the caspase 4/caspase 11–GSDMD pathway [4]. M1 macrophage infiltration also induces chondrocyte hypertrophy through upregulation of COL10A1 [28]. During the implantation process of tissue-engineered cartilage, infiltration of M1 macrophage may occur, leading to the release of proinflammatory mediators such as IL-1, IL-6, TNF-α, and EVs [4, 6, 7]. These mediators inhibit the formation of mature cartilage and promote cartilage degradation metabolism. Therefore, it is crucial to enhance the resistance of remaining ear chondrocytes to M1 macrophage infiltration to facilitate tissue-engineered cartilage formation.

EVs, as lipid-bound nanoparticles, can transport therapeutic molecules to specific cells and have been utilized in aesthetic, plastic, and reconstructive surgery for promoting tissue regeneration, including bone regeneration [29], wound healing [30, 31], treatment of skin diseases [32], medical aesthetics [33], angiogenesis [34], inflammation regulation [35], fat transplantation [36], hair regeneration [37], and peripheral nerve regeneration [38]. EVs derived from hADSCs have shown effectiveness in enhancing tissue-engineered cartilage regeneration [19]. However, it remains unknown whether these hADSCs-derived EVs can exhibit the same therapeutic effects on tissue-engineered cartilage in a microenvironment infiltrated by M1 macrophage. Additionally, the penetration capability of natural hADSCs-derived EVs through dense matrix layers and their specific targeting of chondrocytes are limited. These limitations impede the clinical application of hADSCs-derived EVs in tissue-engineered cartilage regeneration.

In this study, we formulated a bioink by blending porous GelMA hydrogels with remaining ear chondrocytes and EVs for fabricating tissue-engineered cartilage hydrogels. GelMA hydrogels are widely used in various biomedical applications due to their biocompatibility, biodegradability, and noncytotoxic properties. They possess similar characteristics to key components of the native extracellular matrix, such as cell-adhesive and matrix metalloproteinase responsive peptide motifs, promoting cell proliferation and spreading. Chondrocytes can be evenly encapsulated within GelMA hydrogel matrices, ensuring excellent cell stability and viability. The GelMA hydrogel solution laden with chondrocytes can be extruded at various concentrations to create different forms of tissue-engineered cartilage hydrogels. Moreover, numerous studies have shown that GelMA hydrogels serve as effective carriers for EVs, protecting them from degradation with their excellent porosity and mesh structure, thereby promoting tissue repair and regeneration in various tissues. Various types of biomaterial scaffolds have emerged as promising options for tissue-engineered cartilage regeneration. Natural biomaterials such as alginate, pluronic acid, hyaluronic acid, chitosan, and collagen derivatives, similar to GelMA hydrogels, exhibit exceptional biocompatibility and biodegradability. GelMA hydrogels differ from natural biomaterials in that their mechanical strength, degradation rate, porosity, and meshwork can be enhanced by increasing the photocrosslinking time and hydrogel concentration. Synthetic polymers like polyglycolic acid, polylactic acid, and polycaprolactone are commonly used for cartilage tissue scaffolds due to their excellent plasticity and mechanical properties. However, these synthetic polymers may cause severe foreign body reactions, hindering cartilage matrix biosynthesis. While GelMA hydrogels are biocompatible with ear chondrocytes and hADSCs-derived EVs, the individual components of the hydrogels do not accurately replicate the cartilage-specific microenvironment, and their inadequate mechanical stability compromises the fidelity of the 3D morphology. Therefore, future research should investigate hybrid materials that combine natural and synthetic components, such as polycaprolactone, methacrylate-modified acellular cartilage matrix, or bacterial nanocellulose, to solve these challenges.

We introduce a quick and innovative hADSCs spheroids-based pipeline to collect and isolate CHP-EVs. By employing the three-dimensional dynamic culture technology and a harvesting method, we were able to significantly reduce the culture space, experimental time, and expenses through a scale-up strategy. Our findings demonstrate that hADSCs spheroids outperform hADSCs microspheres in terms of increasing EVs yields and loading multiple miRNAs with chondrogenesis potential into the lumen of EVs. As a result, we select the hADSCs spheroids-based pipeline for the production of clinical-grade EVs for tissue-engineered cartilage formation. Furthermore, we have enhanced the targeting ability of these EVs by expressing CHP on their surface membrane, specifically for chondrocytes. We have observed that CHP-EVs can efficiently penetrate dense biomaterial scaffolds and deliver functional miRNAs to remaining ear chondrocytes, even in the presence of M1 macrophage infiltration. CHP-EVs exhibit more significant therapeutic effects on improving the fate of remaining ear chondrocytes and promoting tissue-engineered cartilage formation. Overall, our study has made several novel discoveries.

First, we made significant progress in the mass production of EVs through the implementation of a scale-up strategy using hADSCs spheroids. This strategy has led to a substantial reduction in the required culture space, experimental time, and expenses. Currently, there is a growing focus on creating in vivo-like conditions for cell culture. The development and utilization of various three-dimensional cell culture models have attracted significant interest in the field of cell biology research. This is particularly critical in the study of EVs, where recent investigations comparing EVs derived from two-dimensional and three-dimensional cultures have demonstrated that culture conditions have a significant impact on the properties of EVs, including their size, cargo, therapeutic potential, secretion efficiency, and drug delivery capabilities. Notably, three-dimensional culture has been shown to be more advantageous than two-dimensional culture in terms of the abovementioned properties [16–18]. However, the lack of convenient methods for obtaining EVs from three-dimensional cell cultures remains a bottleneck. Biomaterial scaffold-based three-dimensional cell culture platforms have demonstrated their efficiency in EVs production and loading of beneficial functional molecules. Nonetheless, the gel-like structure of many three-dimensional scaffolds is difficult to dissolve, which may impede the efficient collection of EVs and cells encapsulated within these scaffolds for downstream analyses. Therefore, new three-dimensional culture models for EVs production and isolation are urgently needed. Herein, we first introduced two novel types of three-dimensional culture platforms for the culture of hADSCs, namely, a spheroid-based pipeline and a GelMA microsphere-based pipeline, using a harvesting method. We successfully isolated EVs from these two pipelines, namely, hADSCs spheroids-EVs and hADSCs microspheres-EVs, for simplicity. In our previous study, only 1.8–2.4 μg of EVs were obtained from 1 mL of cell culture supernatant in traditional 2D culture conditions [39]. The yield of EVs from hADSCs spheroids and hADSCs microspheres was significantly higher compared to EVs cultured in 2D culture dishes. The EVs yield of hADSCs spheroids was approximately three times higher than that of hADSCs microspheres. EVs obtained from hADSCs spheroids were much easier and more efficient to isolate compared to those obtained from hADSCs microspheres in terms of both labor and time. Furthermore, we discovered increased expression of chondrogenesis-related miRNAs in EVs derived from hADSCs spheroids compared to those derived from hADSCs microspheres. In our study, we observed that some hADSCs detached from the surface of GelMA microspheres when cultured in a miniaturized wave bioreactor. This indicates that GelMA microspheres exhibit poor adhesive properties and limited matrix-based and cell-cell interactions for hADSC growth. The large specific surface area of GelMA microspheres enables hADSCs to proliferate and migrate, leading to rapid senescence of hADSCs. In contrast, hADSCs in 3D spheroids exhibit poor proliferation due to being in cell cycle arrest. Cellular senescence is also reduced in cell spheroids due to the improved preservation of quiescent stem cells. [40]. Prior research has indicated that the three-dimensional aggregation culture of MSCs can maintain the primitive stem cell phenotype, decrease cellular senescence, and enhance therapeutic benefits by secreting growth factors and cytokines [41]. Additionally, we observed that the growth pattern of hADSCs spheroids is conducive to enhancing cell-cell interactions and extracellular matrix enrichment. This is evidenced by the close aggregation of hADSCs cultured in hADSCs spheroids under defined shear stress using a miniaturized wave bioreactor. The metabolic reconfiguration and stress response in cell spheroids likely induce the activation of broad signaling pathways and play a central role in reacquisition of primitive MSCs phenotypic properties [40, 42]. Overall, the differences in adhesion, growth pattern, cell‒cell interactions, matrix properties and associated intracellular signaling pathways between hADSCs spheroids and hADSCs microspheres may affect cell behavior, growth, EVs production, and miRNA profiles.

Second, there were 11 highly differentially expressed miRNAs between hADSCs spheroids-EVs and hADSCs microspheres-EVs. Among them, nine miRNAs were associated with chondrogenesis, including hsa-miR-486-5p, hsa-miR-423-5p, hsa-miR-92a-3p, hsa-miR-122-5p, hsa-miR-223-3p, hsa-miR-320a, hsa-miR-126-3p, hsa-miR-25-3p, and hsa-miR-146b-5p. The upregulation of eight miRNAs (hsa-miR-486-5p, hsa-miR-423-5p, hsa-miR-92a-3p, hsa-miR-122-5p, hsa-miR-223-3p, hsa-miR-320a, hsa-miR-126-3p, and hsa-miR-25-3p) and the downregulation of hsa-miR-146b-5p within hADSCs spheroids-derived EVs were reported to improve fate of chondrocytes and promote chondrogenesis. To our knowledge, hADSCs-derived EVs containing miR-486-5p showed superior effects in modulating chondrocyte homeostasis in osteoarthritis by inhibiting endoplasmic reticulum stress, alleviating chondrocyte apoptosis, promoting matrix regeneration, and regulating macrophage polarization compared to direct administration of miR-486-5p or miR-486-5p-overexpressing hADSCs [43]. Li et al. [44] reported that human umbilical cord MSCs-derived EVs contain five highly enriched miRNAs: has-miR-122-5p, has-miR-148a-3p, has-miR-486-5p, has-miR-let-7a-5p, and has-miR-100-5p. These miRNAs primarily targeted genes in the PI3K-Akt signaling pathway, which could play a mechanistic role in alleviating cartilage degradation during the progression of osteoarthritis and promoting the polarization of M2 macrophage. This suggested that these EVs had significant potential for immunomodulation. Upregulation of miR-423-5p inhibited chondrocyte inflammation and represented a potential therapeutic target for osteoarthritis [45]. Upregulation of miR-92a-3p exhibited pro-chondrogenic and chondroprotective effects in osteoarthritis treatment through targeting SMAD6/7 [46]. Mao et al. [47] found that exosomal miR-92a-3p from human MSCs promoted chondrocyte proliferation and expression of matrix genes in both MSCs and primary human chondrocytes affected by osteoarthritis by directly targeting WNT5A. Upregulation of miR-92a-3p also regulated cartilage development and maintains homeostasis by directly targeting HDAC2 in both MSCs and primary human chondrocytes affected by osteoarthritis [48]. Additionally, miR-92a-3p downregulated the expression of ADAMTS-4/5 induced by IL-1β in chondrogenic hMSCs and human chondrocytes, thereby inhibiting the progression of osteoarthritis [49]. Upregulated miR-122-5p enhanced the chondrogenic differentiation of MSCs and promoted cartilage regeneration by activating the Wnt1/β-catenin pathway [50]. Upregulated miR-223-3p inhibited apoptosis and the inflammatory response in chondrocytes induced by IL-1β by directly targeting NLRP3 [51]. By targeting the DAZAP1 and MAPK pathways, upregulated miR-320a improved IL-1β-induced osteoarthritis by enhancing chondrocyte proliferation, reducing apoptosis, and decreasing inflammatory response [52]. The miR-320a also played a role in protecting cartilage against degeneration by regulating the protein expression levels of BMI-1 and RUNX2 in chondrocytes [53]. Upregulated miR-126-3p enhanced the viability and migration of human osteoarthritic chondrocytes stimulated by IL-1β, while reducing apoptosis and promoting chondrogenesis [54]. Zhou et al. [55] demonstrated that EVs delivered miR-126-3p to promote the proliferation and migration of chondrocytes, inhibit apoptosis and inflammation, and prevent cartilage degradation. Loading miR-25-3p into EVs has been shown to alleviate pyroptosis in chondrocytes in knee osteoarthritis by inhibiting the transcription of CPEB1 [56]. Furthermore, miR-25-3p promoted chondrocytes proliferation and inhibited chondrocytes apoptosis by negatively regulating IGFBP7 [57]. Ren et al. [58] also reported that miR-25-3p enhanced cell viability and suppressed apoptosis of IL-1β-stimulated chondrocytes by directly inhibiting JPX. The downregulation of miR-146b-5p was beneficial for controlling the inflammatory microenvironment of osteoarthritis and promoting chondrogenesis [59]. These findings suggest that hADSCs spheroids-EVs may deliver chondrogenesis-associated miRNAs to remaining ear chondrocytes, thereby promoting tissue-engineered cartilage regeneration.

Third, the penetration of natural EVs without CHP modification into the densely packed GelMA hydrogels and their specific targeting of the remaining ear chondrocytes prove to be challenging. The encapsulated EVs easily diffuse out of the hydrogels, resulting in a short residence time within the hydrogels. All of these factors contribute to the difficulty of achieving chondrocyte-targeted drug delivery. The engineered surface membrane modifications of EVs aim to enhance their capacity of delivering miRNAs. This is achieved by genetically engineering or chemically modifying/bioconjugating the lipid bilayers of EVs to express targeting peptides or functional proteins. The lipid bilayers of EVs originate from the cell membranes of their parent cells. Therefore, it is possible to modify the surface membrane of EVs through genetic engineering or chemical modification of their parent cells. To achieve this, fusion gene vectors can be constructed to encode membrane proteins associated with EVs, such as LAMP2B [60, 61], CD63 [62, 63], CD9 [64, 65], or a mutant form of Nef (Nef^mut^) [66]. The targeting peptides or ligands, such as ischemic myocardium targeting peptide (IMTP) [60], RVG peptide [61], the TNF-α binding domain of human TNF receptor-1 (hTNFR1) [62], or the RNA binding protein HuR [64], can be coexpressed on the membrane surface of specific EVs. This can be accomplished by creating fusion gene vectors and then transferring them into parent cells. During the process of EVs biosynthesis, the engineered EVs efficiently accumulate EVs-associated membrane proteins in multivesicular bodies. Subsequently, coexpressing EV-associated membrane proteins and targeting peptides/ligands are secreted. In this study, we engineered CHP-EVs and utilized them for tissue-engineered cartilage regeneration in a microenvironment infiltrated by M1 macrophage. The CHP-LAMP2B vector encoded a glycosylation sequence (GNSTM), a CHP sequence (DWRVIIPPRPSA), and a glycine-serine spacer at the N-terminus of the LAMP2B protein. Since the N-terminus of LAMP2B protrudes from the surface membrane of EVs, fusing a targeting peptide CHP at this end does not affect the expression and function of LAMP2B, allowing for the generation of CHP-EVs. An additional GFP tag was fused to downstream of CHP + LAMP2B for fluorescence labeling. ADSCs transfected with the CHP + LAMP2B vector exhibit cytosolic GFP fluorescence, indicating successful expression of CHP + LAMP2B. Western blot analysis confirms the overexpression of LAMP2B in ADSCs transfected with the CHP + LAMP2B vector. CHP-EVs, designed as a targeted drug delivery system in tissue-engineered cartilage, exhibit the following characteristics: (1) exceptional tissue penetration capability through densely packed hydrogel scaffolds, resulting in reduced diffusion and prolonged localization within the hydrogel scaffolds; (2) the comprehensive benefits of EVs derived from hADSCs; and (3) specific delivery of chondrogenesis-associated miRNAs to remaining ear chondrocytes. One shortcoming of our study is that the overexpressed gene (i.e. CHP + LAMP2B) is located upstream of the fluorescent protein, and excessively long gene fragments or high-GC fragments may decrease the intensity of the downstream fluorescent protein, resulting in weaker fluorescence compared to the NC plasmid group.

To date, numerous studies have demonstrated the efficacy of ADSC-derived EVs in enhancing cartilage formation and repairing damaged cartilage. For instance, ADSC-derived EVs released from porous GelMA hydrogel-based 3D cultures have been shown to facilitate the formation of tissue-engineered hyaline and elastic cartilage by delivering functional miRNAs to chondrocytes [19, 39]. ADSCs-derived EVs have shown potential in alleviating osteoarthritis by inhibiting the degradation of the cartilage extracellular matrix [67]. Additionally, these EVs could improve cartilage injury by promoting chondrocyte proliferation and autophagy [68]. Tropoelastin-pretreated ADSCs-derived EVs could maintain the chondrocyte phenotype, enhance cartilage extracellular matrix synthesis and promote repair of damaged cartilage [69]. EVs derived from hypoxia-cultured hADSVs alleviated articular chondrocyte inflammaging and posttraumatic osteoarthritis progression by regulating cellular oxidative stress and attenuating cell senescence [70]. Wang et al. [43] reported that ADSCs-derived EVs could inhibit ER stress, alleviate chondrocytes apoptosis and promote matrix regeneration for damaged cartilage repair. Additionally, kartogenin-pretreated ADSCs-derived EVs could upregulate the expression of chondrogenic differentiation-related genes and downregulate the expression of chondrolysis-related genes [71]. In our study, we utilized CHP-EVs to promote tissue-engineered cartilage formation through modified strategies. First, hADSCs spheroids cultured in a 3D dynamic bioreactor were used to enhance the production and promote the chondrogenesis potential of EVs by loading higher levels of chondrogenesis-associated miRNAs. Second, a harvesting method was used to efficiently collect culture supernatants with high EVs concentrations. Third, CHP was used to modify the surface membrane of EVs. Overall, we found that CHP-EVs were more easily captured by remaining ear chondrocytes in vitro and in vivo, leading to enhanced cell proliferation, cell cycle regulation, decreased cell apoptosis, and increased cartilage matrix biosynthesis in the M1 macrophage-infiltrated microenvironment, compared to LAMP2B-EVs and NC-EVs lacking the CHP sequence on their surface. Similarly, Pi et al. [16] developed a nonviral CHP-modified vector for targeted therapy of cartilage disorders by covalently combining polyethylenimine and CHP. The CHP-modified vector exhibited higher concentrations in cartilage and was specifically internalized by chondrocytes, in contrast to a randomly scrambled peptide (SP)-modified vector. Chongchai et al. [17] reported that CHP could efficiently and selectively deliver transgene expression to pathogenic chondrocytes. Hu et al. [18] developed a CHP-modified PEGylated polyamidoamine nanocarrier that showed no toxicity towards cartilage or major organs. Targeting and penetration evaluations demonstrated that CHP-modified PEGylated PAMAM conjugates could effectively penetrate cartilage tissue and specifically deliver therapeutic agents to chondrocytes for the alleviation of osteoarthritis. The inflammatory microenvironment associated with M1 macrophage infiltration is similar to that of osteoarthritis, and we hypothesize that CHP-EVs may have a protective effect on repairing damaged cartilage. Further investigations should be conducted to explore the role of CHP-EVs in repairing and regenerating damaged cartilage. Our study revealed that CHP-EVs exhibit enhanced efficacy in improving chondrocyte fate and promoting the tissue-engineered cartilage formation, primarily attributed to the surface membrane modification of CHP. CHP not only enhances the uptake of EVs derived from ADSCs by chondrocytes but also prolongs the functionality of these EVs by reducing diffusion and increasing their localization within hydrogel scaffold. Additionally, we hypothesize that different transfection vectors for ADSCs (i.e., CHP + LAMP2B vector and LAMP2B vector) may influence the miRNA profiles of CHP-EVs and LAMP2B-EVs to some extent, subsequently affecting their biological functions in cartilage formation. Further studies are needed to compare the miRNA profiles of CHP-EVs and LAMP2B-EVs and determine the presence of differentially expressed miRNAs.

In this study, an animal model was used to validate the efficacy of CHP-EVs in repairing tissue-engineered cartilage damaged by activated M1 macrophage infiltration. Instead of implanting tissue-engineered cartilage samples into the subcutaneous sites of microtia patients, BALB/c nude mice were chosen for implanting the tissue-engineered cartilage samples to simulate in vivo growth. This model can help researchers study the role of CHP-EVs in remaining ear chondrocytes and simultaneously avoid immunological rejection. However, the subcutaneous physiological environment of nude mice differs from human. To mimic the microenvironment of tissue-engineered cartilage damaged by activated M1 macrophage, an equal number of freshly activated M1 macrophage was directly injected into multiple sites of the tissue-engineered cartilage samples every 3 days to ensure sufficient infiltration surrounding the tissue-engineered cartilage, following a similar approach to the study conducted by Wang et al. [72], who employed the local injection of macrophage into targeting adipose tissues. This injection technique is challenging and may easily damage tissue-engineered cartilage samples. Moreover, the number of M1 macrophage injected may not accurately represent the actual number of M1 macrophage infiltrating the target sites. Other methods for assessing M1 macrophage infiltration have been reported. For instance, M1 macrophage can infiltrate tissues through vein injection [73]. However, this approach may induce acute inflammation and even mortality in experimental animals. The portal vein injection method lacks the ability to controllably retain M1 macrophage at the desired target sites. Additionally, Guerra et al. [74] reported a method of M1 macrophage hydrogels where activated M1 macrophage was loaded into a thiolated gelatin poly (ethylene glycol) (Gel-PEG-Cys) and poly (ethylene glycol) diacrylate cross-linked hydrogel. The M1 macrophage hydrogel was then injected adjacent to the target sites on a weekly basis to mimic the effect of M1 macrophage infiltration. While this method allows for the controllable retention of M1 macrophage at specific target sites, it does not promote direct interaction between M1 macrophages and the target sites. Additionally, the injected hydrogel accumulates in the experimental animals and may result in a body burden.

While this study demonstrated the effectiveness of CHP-EVs in tissue-engineered cartilage formation, there are still some limitations. First, it is necessary to compare the miRNA profiles of hADSCs spheroids and hADSCs GelMA microspheres and further analyze the miRNA profiles of their corresponding EVs. This will help to understand how the culture microenvironment influences the production of EVs and the functional molecule loading, thereby affecting the paracrine functions of parental hADSCs. Second, within the lumen of hADSCs spheroids-EVs, we identified eight upregulated miRNAs and one downregulated miRNA, each of which plays a distinct role in cartilage regeneration. It is speculated that hADSCs spheroids-derived EVs may influence the remaining ear chondrocytes through integrated regulatory mechanisms. However, the key miRNA is unknown in our study. Third, our study only focuses on the small-scale production of CHP-EVs for basic research using a miniaturized wave bioreactor, which is inadequate for clinical applications. Efforts are underway to enhance scalability and facilitate clinical translation. We plan to utilize a full-scale wave bioreactor or vertical wheel bioreactor for the large-scale production of CHP-EVs. The translational potential depends on the large-scale culture system for hADSCs spheroids, and it is essential to thoroughly characterize the secreted CHP-EVs in the bioreactors. Demonstrating the consistency of the miRNA cargo profiles of CHP-EVs for each bioreactor run is crucial for quality control. Additionally, this study should optimize several experimental factors to enhance the production and function of CHP-EVs. For example, it is essential to determine the minimum effective dosage of CHP-EVs for tissue-engineered cartilage regeneration, which may be achieved by reducing the dosage and increasing the frequency of administration. The number of hADSCs used in hADSCs spheroids should also be optimized to ensure optimal cell viability and EVs production. Finally, we collect cell supernatant using a harvesting method every 24 h. To streamline this process and save time and effort, we should develop an automated procedure for collecting cell supernatant. Fourth, gene expression involves transcription and translation at the mRNA and protein levels respectively. Transcription and translation in eukaryotic gene expression occur at different times and locations. Following transcription, there are processes such as posttranscriptional processing, degradation of transcription products, translation, posttranslational processing, and modification that take place at various levels. Additionally, mRNA and proteins exhibit different half-lives. While mRNAs are prone to degradation and can persist for a short period in tissues, the proteins translated from these mRNAs are inherently more stable. As a result, the transcription level and translation level may not be entirely consistent or show a linear correlation. In our study, we find that CHP-EVs strongly regulate BCL-2 and BAX at the transcriptional level but not at the translation level compared with LAMP2B-EVs.

## Conclusions

In conclusion, ADSCs spheroids-EVs enriched with various chondrogenesis-associated miRNAs significantly improve the fate of remaining ear chondrocytes and promote tissue-engineered cartilage formation through integrated signaling pathways. A genetic engineering strategy is developed to modify the surface membrane, known as CHP-EVs, allowing for targeted delivery to chondrocytes and efficient penetration into hydrogel scaffolds. This chondrocyte-targeted, EVs-mediated drug delivery system offers a promising approach for tissue-engineered cartilage formation.

### Supplementary Information

Additional file1. Antibodies for WB and immunohistochemically staining.

Additional file2. Primer sequences for qRT-PCR.

## Data Availability

All data generated or analyzed during this study are included in this published article.

## References

[1] Zhou G, Jiang H, Yin Z, Liu Y, Zhang Q, Zhang C (2018). In vitro regeneration of patient-specific ear-shaped cartilage and its first clinical application for auricular reconstruction. EBioMedicine.

[2] Satoh T, Kidoya H, Naito H, Yamamoto M, Takemura N, Nakagawa K (2013). Critical role of trib1 in differentiation of tissue-resident M2-like macrophages. Nature.

[3] Yoshida S, Nishitani K, Yoshitomi H (2023). Knee alignment correction by high tibial osteotomy reduces symptoms and synovial inflammation in knee osteoarthritis accompanied by macrophage phenotypic change from M1 to M2. Arthritis Rheumatol.

[4] Ebata T, Terkawi MA, Kitahara K, Yokota S, Shiota J, Nishida Y (2023). Noncanonical pyroptosis triggered by macrophage-derived extracellular vesicles in chondrocytes leading to cartilage catabolism in osteoarthritis. Arthr Rheumatol.

[5] Zeng J, Jia L, Wang D, Chen Z, Liu W, Yang Q (2022). Bacterial nanocellulose-reinforced gelatin methacryloyl hydrogel enhances biomechanical property and glycosaminoglycan content of three-dimensional-bioprinted cartilage. Int J Bioprint.

[6] Zhang H, Lin C, Zeng C, Wang Z, Wang H, Lu J (2018). Synovial macrophage M1 polarisation exacerbates experimental osteoarthritis partially through R-spondin-2. Ann Rheum Dis.

[7] Yan Y, Lu A, Dou Y, Zhang Z, Wang XY, Zhai L (2023). Nanomedicines reprogram synovial macrophages by scavenging nitric oxide and silencing CA9 in progressive osteoarthritis. Adv Sci.

[8] Kalluri R, LeBleu VS (2020). The biology, function, and biomedical applications of exosomes. Science.

[9] O'Brien K, Breyne K, Ughetto S, Laurent LC, Breakefield XO (2020). RNA delivery by extracellular vesicles in mammalian cells and its applications. Nat Rev Mol Cell Biol.

[10] Zhao S, Xiu G, Wang J, Wen Y, Lu J, Wu B (2023). Engineering exosomes derived from subcutaneous fat MSCs specially promote cartilage repair as miR-199a-3p delivery vehicles in osteoarthritis. J Nanobiotechnol.

[11] Mianehsaz E, Mirzaei HR, Mahjoubin-Tehran M, Rezaee A, Sahebnasagh R, Pourhanifeh MH (2019). Mesenchymal stem cell-derived exosomes: a new therapeutic approach to osteoarthritis?. Stem Cell Res Ther.

[12] Li Q, Yu H, Sun M, Yang P, Hu X, Ao Y, Cheng J (2021). The tissue origin effect of extracellular vesicles on cartilage and bone regeneration. Acta Biomater.

[13] Han M, Yang H, Lu X, Li Y, Liu Z, Li F (2022). Three-dimensional-cultured MSC-derived exosome-hydrogel hybrid microneedle array patch for spinal cord repair. Nano Lett.

[14] Yuan X, Sun L, Jeske R, Nkosi D, York SB, Liu Y (2022). Engineering extracellular vesicles by three-dimensional dynamic culture of human mesenchymal stem cells. J Extracell Vesicles.

[15] Kyykallio H, Faria AVS, Hartmann R, Capra J, Rilla K, Siljander PR (2022). A quick pipeline for the isolation of 3D cell culture-derived extracellular vesicles. J Extracell Vesicles.

[16] Pi Y, Zhang X, Shi J, Zhu J, Chen W, Zhang C (2011). Targeted delivery of non-viral vectors to cartilage in vivo using a chondrocyte-homing peptide identified by phage display. Biomaterials.

[17] Chongchai A, Waramit S, Wongwichai T, Kampangtip J, Phitak T, Kongtawelert P (2021). Targeting human osteoarthritic chondrocytes with ligand directed bacteriophage-based particles. Viruses.

[18] Hu Q, Chen Q, Yan X (2018). Chondrocyte affinity peptide modified PAMAM conjugate as a nanoplatform for targeting and retention in cartilage. Nanomedicine.

[19] Chen J, Huang T, Liu R, Wang C, Jiang H, Sun H (2022). Congenital microtia patients: the genetically engineered exosomes released from porous gelatin methacryloyl hydrogel for downstream small RNA profiling, functional modulation of microtia chondrocytes and tissue-engineered ear cartilage regeneration. J Nanobiotechnol.

[20] Peng S, Yan Y, Li R, Dai H, Xu J (2021). Extracellular vesicles from M1-polarized macrophages promote inflammation in the temporomandibular joint via miR-1246 activation of the Wnt/β-catenin pathway. Ann N Y Acad Sci.

[21] Bi Y, Qiao X, Liu Q, Song S, Zhu K, Qiu X (2022). Systemic proteomics and miRNA profile analysis of exosomes derived from human pluripotent stem cells. Stem Cell Res Ther.

[22] Théry C, Witwer KW, Aikawa E, Alcaraz MJ, Anderson JD, Andriantsito-haina R (2018). Minimal information for studies of extracellular vesicles 2018 (MISEV2018): a position statement of the international society for extracellular vesicles and update of the MISEV2014 guidelines. J Extracell Vesicles.

[23] Hung ME, Leonard JN (2015). Stabilization of exosome-targeting peptides via engineered glycosylation. J Biol Chem.

[24] Lopa S, Leijs MJ, Moretti M (2015). Arthritic and non-arthritic synovial fluids modulate IL10 and IL1RA gene expression in differentially activated primary human monocytes. Osteoarthr Cartil.

[25] Cui SH, Yan Y, Lu A (2024). Nanomedicines promote cartilage regeneration in osteoarthritis by synergistically enhancing chondrogenesis of mesenchymal stem cells and regulating inflammatory environment. ACS Nano.

[26] Li H, Yuan Y, Zhang L, Xu C, Xu H, Chen Z (2024). Reprogramming macrophage polarization, depleting ROS by astaxanthin and thioketal-containing polymers delivering rapamycin for osteoarthritis treatment. Adv Sci.

[27] Bondeson J, Wainwright SD, Lauder S, Amos N, Hughes CE (2006). The role of synovial macrophages and macrophage-produced cytokines in driving aggrecanases, matrix metalloproteinases, and other destructive and inflammatory responses in osteoarthritis. Arthritis Res Ther.

[28] Qian Y, Chu G, Zhang L (2024). M2 macrophage-derived exosomal miR-26b-5p regulates macrophage polarization and chondrocyte hypertrophy by targeting TLR3 and COL10A1 to alleviate osteoarthritis. J Nanobiotechnol.

[29] Bari E, Roato I, Perale G (2021). Biohybrid bovine bone matrix for controlled release of mesenchymal stem/stromal cell lyosecretome: a device for bone regeneration. Int J Mol Sci.

[30] Qian L, Pi L, Fang BR, Meng XX (2021). Adipose mesenchymal stem cell-derived exosomes accelerate skin wound healing via the lncRNA H19/miR-19b/SOX9 axis. Lab Invest.

[31] Shi R, Jin Y, Hu W (2020). Exosomes derived from mmu_circ_0000250- modified adipose-derived mesenchymal stem cells promote wound healing in diabetic mice by inducing miR-128-3p/SIRT1-mediated autophagy. Am J Physiol Cell Physiol.

[32] Shin KO, Ha DH, Kim JO (2020). Exosomes from human adipose tissue- derived mesenchymal stem cells promote epidermal barrier repair by inducing de novo synthesis of ceramides in atopic dermatitis. Cells.

[33] Liang JX, Liao X, Li SH (2020). Antiaging properties of exosomes from adipose- derived mesenchymal stem cells in photoaged rat skin. Biomed Res Int.

[34] Lopatina T, Bruno S, Tetta C (2014). Platelet-derived growth factor regulates the secretion of extracellular vesicles by adipose mesenchymal stem cells and enhances their angiogenic potential. Cell Commun Signal.

[35] Heo JS, Lim JY, Yoon DW (2020). Exosome and melatonin additively attenuates inflammation by transferring miR-34a, miR-124, and miR-135b. Biomed Res Int.

[36] Hao X, Guo Y, Wang R (2021). Exosomes from adipose-derived mesenchymal stem cells promote survival of fat grafts by regulating macrophage polarization via let-7c. Acta Biochim Biophys Sin.

[37] Wu J, Yang Q, Wu S (2021). Adipose-derived stem cell exosomes promoted hair regeneration. Tissue Eng Regen Med.

[38] Chen J, Ren S, Duscher D (2019). Exosomes from human adipose-derived stem cells promote sciatic nerve regeneration via optimizing Schwann cell function. J Cell Physiol.

[39] Chen JG, Zhang EC, Wan YY (2024). Engineered hsa-miR-455-3p-abundant extracellular vesicles derived from three-dimensional-cultured adipose mesenchymal stem cells for tissue-engineering hyaline cartilage regeneration. Adv Healthc Mater.

[40] Bijonowski BM, Yuan X, Jeske R, Li Y, Grant SC (2020). Cyclical aggregation extends in vitro expansion potential of human mesenchymal stem cells. Sci Rep.

[41] Bijonowski BM, Fu Q, Yuan X, Irianto J, Li Y, Grant SC, Ma T (2020). Aggregation-induced integrated stress response rejuvenates culture-expanded human mesenchymal stem cells. Biotechnol Bioeng.

[42] Liu Y, Muñoz N, Tsai AC, Logan TM, Ma T (2017). Metabolic reconfiguration supports reacquisition of primitive phenotype in human mesenchymal stem cell aggregates. Stem Cell.

[43] Wang Y, Fan A, Lu L, Pan Z, Ma M, Luo S (2022). Exosome modification to better alleviates endoplasmic reticulum stress induced chondrocyte apoptosis and osteoarthritis. Biochem Pharmacol.

[44] Li K, Yan G, Huang H, Zheng M, Ma K, Cui X (2022). Anti-inflammatory and immunomodulatory effects of the extracellular vesicles derived from human umbilical cord mesenchymal stem cells on osteoarthritis via M2 macrophages. J Nanobiotechnol.

[45] Chen K, Fang H, Xu N (2020). LncRNA LOXL1-AS1 is transcriptionally activated by JUND and contributes to osteoarthritis progression via targeting the miR-423-5p/KDM5C axis. Life Sci.

[46] Zheng C, Hoshi K, Hikita A (2023). miR-92a-3p-inspired shRNA exhibits pro-chondrogenic and chondrocyte protective effects in osteoarthritis treatment through targeting SMAD6/7. J Bone Miner Metab.

[47] Mao G, Zhang Z, Hu S, Zhang Z, Chang Z, Huang Z (2018). Exosomes derived from miR-92a-3p-overexpressing human mesenchymal stem cells enhance chondrogenesis and suppress cartilage degradation via targeting WNT5A. Stem Cell Res Ther.

[48] Mao G, Zhang Z, Huang Z, Chen W, Huang G, Meng F (2017). MicroRNA-92a-3p regulates the expression of cartilage-specific genes by directly targeting histone deacetylase 2 in chondrogenesis and degradation. Osteoarthr Cartil.

[49] Mao G, Wu P, Zhang Z, Zhang Z, Liao W, Li Y, Kang Y (2017). MicroRNA-92a-3p regulates aggrecanase-1 and aggrecanase-2 expression in chondrogenesis and IL-1β-induced catabolism in human articular chondrocytes. Cell Physiol Biochem.

[50] Alahdal M, Huang R, Duan L, Zhiqin D, Hongwei O, Li W, Wang D (2021). Indoleamine 2, 3 dioxygenase 1 impairs chondrogenic differentiation of mesenchymal stem cells in the joint of osteoarthritis mice model. Front Immunol.

[51] Dong HC, Li PN, Chen CJ, Xu X, Zhang H, Liu G (2019). Sinomenine attenuates cartilage degeneration by regulating miR-223-3p/NLRP3 inflammasome signaling. Inflammation.

[52] Mao J, Zhang L (2023). MiR-320a upregulation improves IL-1β-induced osteoarthritis via targeting the DAZAP1 and MAPK pathways. J Orthop Surg Res.

[53] Peng H, Liang D, Li B, Liang C, Huang W, Lin H (2017). MicroRNA-320a protects against osteoarthritis cartilage degeneration by regulating the expressions of BMI-1 and RUNX2 in chondrocytes. Pharmazie.

[54] Li S, Stöckl S, Lukas C, Herrmann M, Brochhausen C, König MA (2021). Curcumin-primed human BMSC-derived extracellular vesicles reverse IL-1β-induced catabolic responses of OA chondrocytes by upregulating miR-126-3p. Stem Cell Res Ther.

[55] Zhou Y, Ming J, Li Y, Li B, Deng M, Ma Y (2021). Exosomes derived from miR-126-3p-overexpressing synovial fibroblasts suppress chondrocyte inflammation and cartilage degradation in a rat model of osteoarthritis. Cell Death Discov.

[56] Wang J, Sun T (2023). Mir-25-3p in extracellular vesicles from fibroblast-like synoviocytes alleviates pyroptosis of chondrocytes in knee osteoarthritis. J Bioenerg Biomembr.

[57] He X, Deng L (2021). Potential of miR-25-3p in protection of chondrocytes: emphasis on osteoarthritis. Folia Histochem Cytobiol.

[58] Ren Z, Tang L, Ding Z, Song J, Zheng H, Li D (2022). Knockdown of lncRNA JPX suppresses IL-1β-stimulated injury in chondrocytes through modulating an miR-25-3p/PPID axis. Oncol Lett.

[59] Jia H, Duan L, Yu P, Zhou Y, Liu R, Wang H (2022). Digoxin ameliorates joint inflammatory microenvironment by downregulating synovial macrophage M1-like-polarization and its-derived exosomal miR-146b-5p/Usp3&Sox5 axis. Int Immunopharmacol.

[60] Wang X, Chen Y, Zhao Z (2018). Engineered exosomes with ischemic myocardium- targeting peptide for targeted therapy in myocardial infarction. J Am Heart Assoc.

[61] Yu Y, Li W, Mao L (2021). Genetically engineered exosomes display RVG peptide and selectively enrich a neprilysin variant: a potential formulation for the treatment of alzheimer’s disease. J Drug Target.

[62] Duong N, Curley K, Brown A (2019). Decoy exosomes as a novel biologic reagent to antagonize inflammation. Int J Nanomed.

[63] Curley N, Levy D, Do MA (2020). Sequential deletion of CD63 identifies topologically distinct scaffolds for surface engineering of exosomes in living human cells. Nanoscale.

[64] Li Z, Zhou X, Wei M (2019). In vitro and in Vivo RNA inhibition by CD9-HuR functionalized exosomes encapsulated with miRNA or CRISPR/dCas9. Nano Lett.

[65] Cheng Q, Dai Z, Shi X (2021). Expanding the toolbox of exosome-based modulators of cell functions. Biomaterials.

[66] Ferrantelli F, Manfredi F, Chiozzini C (2018). DNA vectors generating engineered exosomes potential CTL vaccine candidates against AIDS, hepatitis B, and tumors. Mol Biotechnol.

[67] Yin Z, Qin C, Pan S (2023). Injectable hyperbranched PEG crosslinked hyaluronan hydrogel microparticles containing mir-99a-3p modified subcutaneous ADSCs-derived exosomes was beneficial for long-term treatment of osteoarthritis. Mater Today Bio.

[68] Meng C, Na Y, Han C (2023). Exosomal miR-429 derived from adipose-derived stem cells ameliorated chondral injury in osteoarthritis via autophagy by targeting FEZ2. Int Immunopharmacol.

[69] Meng S, Tang C, Deng M (2023). Tropoelastin-pretreated exosomes from adipose-derived stem cells improve the synthesis of cartilage matrix and alleviate osteoarthritis. J Funct Biomater.

[70] Chang LH, Wu SC, Chen CH (2023). Exosomes derived from hypoxia-cultured human adipose stem cells alleviate articular chondrocyte inflammaging and post-traumatic osteoarthritis progression. Int J Mol Sci.

[71] Xie A, Xue J, Wang Y (2022). Kartogenin induced adipose-derived stem cell exosomes enhance the chondrogenic differentiation ability of adipose-derived stem cells. Dis Mark.

[72] Wang YN, Tang Y, He Z (2021). Slit3 secreted from M2-like macrophages increases sympathetic activity and thermogenesis in adipose tissue. Nat Metab.

[73] Chen YJ, Li GN, Li XJ (2023). Targeting IRG1 reverses the immunosuppressive function of tumor-associated macrophages and enhances cancer immunotherapy. Sci Adv.

[74] Guerra AD, Yeung OWH, Qi X, Kao WJ, Man K (2017). The anti-tumor effects of M1 macrophage-loaded poly (ethylene glycol) and gelatin-based hydrogels on hepatocellular carcinoma. Theranostics.

