# Supplementary material for: A quick and innovative pipeline for producing chondrocyte-homing peptide-modified extracellular vesicles by three-dimensional dynamic culture of hADSCs spheroids to modulate the fate of remaining ear chondrocytes in the M1 macrophage-infiltrated microenvironment
Source: J Nanobiotechnology. 2024 May 30;22:300. doi: 10.1186/s12951-024-02567-5 (PMC11141023; doi:10.1186/s12951-024-02567-5)
Supplement: Supplementary file 1 — Additional file1. Antibodies for WB and immunohistochemically staining. [file 12951_2024_2567_MOESM1_ESM.doc]

**Additional file 1.** Antibodies for WB and immunohistochemistry staining.

| Antibody | Application | Clone | Dilution | Company |
| --- | --- | --- | --- | --- |
| CD63 | WB | Rabbit monoclonal | 1:1000 | abcam |
| CD81 | WB | Rabbit monoclonal | 1:1000 | abcam |
| TSG101 | WB | Rabbit monoclonal | 1:1000 | abcam |
| HSP70 | WB | Rabbit monoclonal | 1:1000 | abcam |
| LAMP2B | WB | Rabbit polyclonal | 1 µg/ml | abcam |
| Collagen II | WB | Rabbit monoclonal | 1:1000 | Invitrogen |
| SOX9 | WB | Rabbit monoclonal | 1:1000 | abcam |
| Collagen I | WB | Rabbit polyclonal | 1:1000 | CST |
| COMP | WB | Rabbit monoclonal | 1:1000 | abcam |
| MMP13 | WB | Rabbit monoclonal | 1:1000 | abcam |
| BCL-2 | WB | Rabbit monoclonal | 1:2000 | abcam |
| BAX | WB | Rabbit monoclonal | 1:1000 | abcam |
| GAPDH | WB | Mouse polyclonal | 1:3000 | Huaxingbio |
| Collagen II | IHC-P | Rabbit polyclonal | 1:100 | abcam |
| SOX9 | IHC-P | Rabbit monoclonal | 1:1000 | abcam |
| Collagen I | IHC-P | Rabbit monoclonal | 1:1500 | abcam |
| COMP | IHC-P | Rabbit monoclonal | 1:2000 | abcam |
| Aggrecan | IHC-P | Mouse monoclonal | 0.05 µg/ml | abcam |
| BCL-2 | IHC-P | Rabbit monoclonal | 1:500 | abcam |
| BAX | IHC-P | Rabbit monoclonal | 1:250 | abcam |
