# Supplementary material for: A quick and innovative pipeline for producing chondrocyte-homing peptide-modified extracellular vesicles by three-dimensional dynamic culture of hADSCs spheroids to modulate the fate of remaining ear chondrocytes in the M1 macrophage-infiltrated microenvironment
Source: J Nanobiotechnology. 2024 May 30;22:300. doi: 10.1186/s12951-024-02567-5 (PMC11141023; doi:10.1186/s12951-024-02567-5)
Supplement: Supplementary file 2 — Additional file2. Primer sequences for qRT-PCR. [file 12951_2024_2567_MOESM2_ESM.doc]

| Gene | Primer sequence (5' to 3') |
| --- | --- |
| *COL2A1* | **F:** TGGACGCCATGAAGGTTTTCT, **R:** TGGGAGCCAGATTGTCATCTC |
| *ACAN* | **F:** ACTCTGGGTTTTCGTGACTCT, **R:** ACACTCAGCGAGTTGTCATGG |
| *COL1A1* | **F:** GAGGGCCAAGACGAAGACATC, **R:** CAGATCACGTCATCGCACAAC |
| *SOX9* | **F:** AGCGAACGCACATCAAGAC, **R:** CTGTAGGCGATCTGTTGGGG |
| *COMP* | **F:** GATCACGTTCCTGAAAAACACG, **R:** GCTCTCCGTCTGGATGCAG |
| *MMP13* | **F:** TCCTGATGTGGGTGAATACAATG, **R:** GCCATCGTGAAGTCTGGTAAAAT |
| *BCL-2* | **F:** GGTGGGGTCATGTGTGTGG, **R:** CGGTTCAGGTACTCAGTCATCC |
| *BAX* | **F:** CCCGAGAGGTCTTTTTCCGAG, **R:** CCAGCCCATGATGGTTCTGAT |
| *GAPDH* | **F:** GGAGCGAGATCCCTCCAAAAT, **R:** GGCTGTTGTCATACTTCTCATGG |

**Additional file 2.** Primer sequences for qRT-PCR.
